# Ultrafast Fragment Screening Using Photo-Hyperpolarized (CIDNP) NMR

**DOI:** 10.1021/jacs.3c01392

**Published:** 2023-05-25

**Authors:** Felix Torres, Matthias Bütikofer, Gabriela R. Stadler, Alois Renn, Harindranath Kadavath, Raitis Bobrovs, Kristaps Jaudzems, Roland Riek

**Affiliations:** † ETH, Swiss Federal Institute of Technology, Laboratory of Physical Chemistry, Vladimir-Prelog-Weg 2, CH-8093 Zürich, Switzerland; ‡ NexMR GmbH, Wiesenstrasse 10A, 8952 Schlieren, Switzerland; § St. Jude Children’s Research Hospital, 262 Danny Thomas Place, Memphis, Tennessee 38105-3678 United States; ∥ Latvian Institute of Organic Synthesis, Aizkraukles street 21, LV-1006 Riga, Latvia

## Abstract

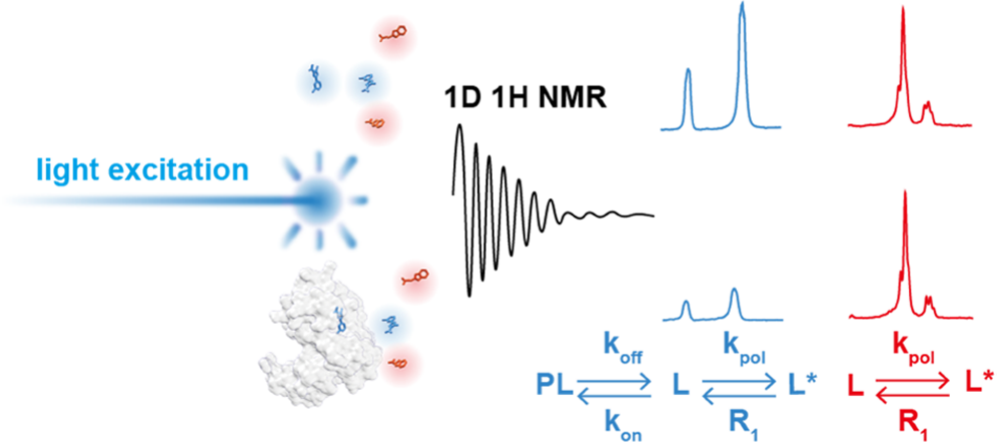

While nuclear magnetic resonance (NMR) is regarded as a reference in fragment-based drug design, its implementation in a high-throughput manner is limited by its lack of sensitivity resulting in long acquisition times and high micromolar sample concentrations. Several hyperpolarization approaches could, in principle, improve the sensitivity of NMR also in drug research. However, photochemically induced dynamic nuclear polarization (photo-CIDNP) is the only method that is directly applicable in aqueous solution and agile for scalable implementation using off-the-shelf hardware. With the use of photo-CIDNP, this work demonstrates the detection of weak binders in the millimolar affinity range using low micromolar concentrations down to 5 μM of ligand and 2 μM of target, thereby exploiting the photo-CIDNP-induced polarization twice: (i) increasing the signal-to-noise by one to two orders in magnitude and (ii) polarization-only of the free non-bound molecule allowing identification of binding by polarization quenching, yielding another factor of hundred in time when compared with standard techniques. The interaction detection was performed with single-scan NMR experiments of a duration of 2 to 5 s. Taking advantage of the readiness of photo-CIDNP setup implementation, an automated flow-through platform was designed to screen samples at a screening rate of 1500 samples per day. Furthermore, a 212 compounds photo-CIDNP fragment library is presented, opening an avenue toward a comprehensive fragment-based screening method.

## Introduction

Nuclear magnetic resonance (NMR) is a powerful tool to monitor and characterize target–ligand interactions. It is able to detect weak and strong affinity binders using various approaches, including saturation transfer difference (STD) experiments detecting interaction on the ligand^1^ or ligand-induced chemical shift perturbation experiments, usually on stable isotope-labeled proteins via two-dimensional (2D) correlation experiments such as heteronuclear single quantum coherence spectroscopy (HSQC) and heteronuclear multiple quantum coherence spectroscopy (HMQC).^2^ It is thereby noted that the NMR experiments are performed simply with the target and ligands in buffer solution without any assay developments usually required, for example, in X-ray crystallography assays or surface plasmon resonance methods.^3−5^ However, NMR suffers from its inherent poor sensitivity yielding several restrictions. First, NMR needs concentrated protein and ligand samples, which not only increases the sample quantity and, correspondingly, the screening price but may also result in protein aggregation, ligand micelle formation, or precipitation. Second, NMR requires long measurement times, between 15 to 30 min per sample.

We present a hyperpolarized NMR method based on photochemically induced dynamic nuclear polarization (photo-CIDNP) to increase the throughput of NMR ligand–target interaction screening by a factor of 10 to 100-fold and reduce the sample concentration by a factor of 10 to 50-fold as we shall see. Photo-CIDNP is one of many other hyperpolarization techniques such as dynamic nuclear polarization (DNP),^6^ para-hydrogen-induced polarization (PHIP),^7^ and signal amplification by reversible exchange (SABRE).^8^ In comparison to the latter techniques, photo-CIDNP presents the unique advantage of inducing hyperpolarization at room temperature in an aqueous solution, and the trigger is simply shining light on the sample.^9,10^ Such conditions are in line with screening-based drug discovery experiments and make photo-CIDNP a good candidate for high-throughput NMR-based small-molecule screening. However, the photo-CIDNP effect has been demonstrated only for approximately 30 molecules.^11−13^ We report in this manuscript an approach to identify small molecules that can be hyperpolarized using photo-CIDNP, the inclusion of these molecules in a library compatible with photo-CIDNP hyperpolarization, and a method to semi-quantitatively identify the binding capacity of a ligand to a protein, which relies on photo-CIDNP quenching upon protein interaction. We applied this approach to select 287 fragment molecules (<300 Da) and screened 212 of them against the proline cis/trans isomerase PIN1 protein, a target relevant in human medicine, in particular in cancer.^14−17^

## Theory and Proof of Concept

Photo-CIDNP was discovered independently by Bargon & Fischer^18^ and Ward & Lawler^19^ in 1967. The effect originates from the formation of radical pairs between a photosensitizer (PS) and a ligand molecule (L), whose recombination, when placed in a magnetic field, depends on the nuclear spin state.^20,21^ The recombined products are chemically identical to the original reactants; however, the spin state will be present in an equilibrium that is out of the Boltzmann equilibrium. The mechanism is well understood and described extensively in the literature.^9^ For the sake of clarity, we summarize the photo-CIDNP effect under its kinetic nature, i.e., as a second-order reaction.
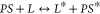
Where PS is the photosensitizer, *L* is the ligand, and PS* and L* are their hyperpolarized states, respectively. As the PS concentration is maintained constant, the photo-CIDNP reaction can be approximated to a pseudo-first-order reaction. Hence, the ligand *L* is polarized into L* with the rate *k*_cidnp_ and relaxes to *L* with the corresponding longitudinal NMR relaxation rate *R*_1_.
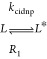
In the presence of a protein (*P*), if and only if the ligand interacts with the protein, the equation set is changed to
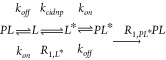


The presence of a binding partner (*P*) introduces two effects: (1) the free ligand concentration (*L*) available to form radical pairs with the PS decreases to form the intermolecular protein–ligand complex (*PL*) and (2) the the relaxation rate *R*_1,PL*_ of the bound hyperpolarized ligand (PL*) increases relative to the relaxation rate of the free hyperpolarized ligand (*R*_1,L*_). Such an increase is not necessarily observed for a thermally polarized system due to the dependency of longitudinal relaxation on spectral density *J*(ω).^22^ However, at out-of-Boltzmann polarization, the contribution of the auto-relaxation term of the bound hyperpolarized ligand to the selective longitudinal relaxation has to be considered, and this term is proportional to *J*(0) and increases with the tumbling time.^23^

Two scenarios are considered; the first is where the relaxation rate of the bound hyperpolarized ligand (*R*_1,PL*_) is much higher than its off-rate (*k*_off_), and the ligand polarization fully goes back to equilibrium during the residence time in the binding pocket. In this case, the longitudinal relaxation of L* is equivalent to *R*_1,L*_ + *k*_on_. The second is where the bound hyperpolarized ligand relaxation rate is slower than the off-rate, and the ligand polarization partially relaxes back to the thermal equilibrium. Considering that primary NMR screenings yield mostly weak binders (affinity from μM to mM) and assuming a *k*_on_ close to the diffusion limit (ca. 10^9^ M^–1^ s^–1^), we presume most *k*_off_ rates to be in the range of 10^6^ to 10^3^ s^–1^ corresponding to residency times of μs to ms. Typical longitudinal relaxation times are in the range of s to high ms, suggesting that the second scenario is present in most cases. Considering that we are in the second scenario with fast exchanging ligand and the free thermally polarized ligand (*L*) is in large excess in comparison to L*,^9,24^ we simplify the equation system to become
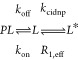
With *R*_1,eff_ = *pR*_1,L*_ + (1 – *p*)*R*_1,PL*_ and *p* is the fraction of unbound ligand, *p* = *L*/(*L* + *PL*). It is important to note that the equilibrium PL* ⇌ L* is established much faster than photo-CIDNP and therefore we consider it to be established at all times.

Therefore, we write eq 1 for the photo-CIDNP polarization build-up
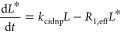
1

As typical photo-CIDNP signal enhancements (20–50-fold) correspond to 0.8–2.0% polarization (L*), we assume stationary conditions where the concentration of *L* is considered constant over time.

Equation 1 is readily solved to obtain
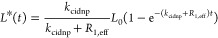
2*L*_0_ is the total free amount of free ligand (*L* + L*), which corresponds as well to *L*_t=0_, the initial concentration of thermally polarized free ligand (*L*).

The first order part of the Taylor expansion of eq 2 shows that the initial regime is governed by the polarization rate *k*_cidnp_ and the initial free ligand concentration (*L*_0_) in further orders, the relaxation term (*R*_1,eff_) influences the ligand polarization (L*).
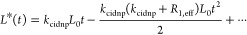
3

At the steady-state regime, which is reached for times greater than 1/(*R*_1,eff_ + *k*_cidnp_), an inspection of eq 2 demonstrates
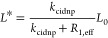
4

**Figure 1:**
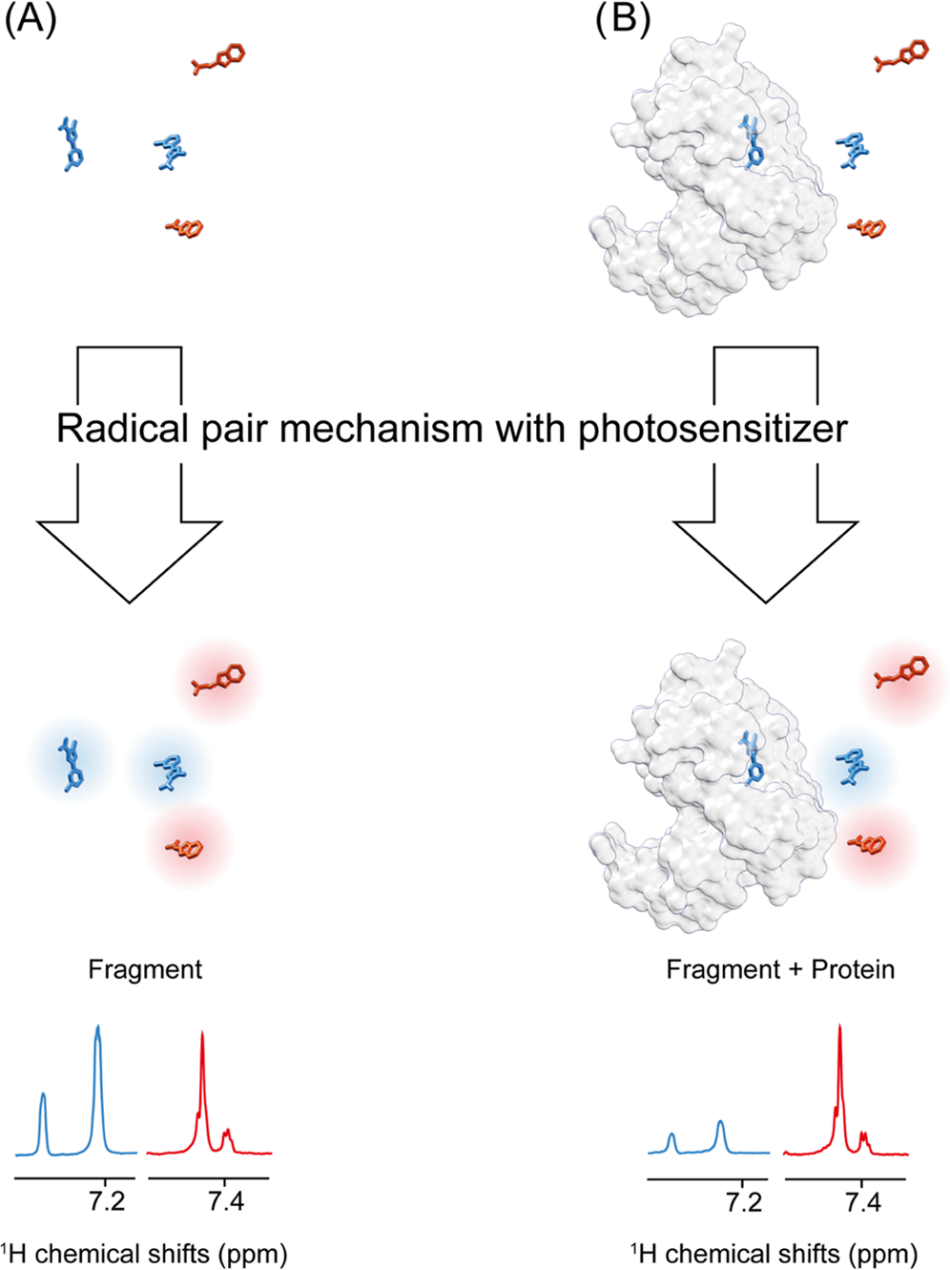
Schematic representation of the photo-CIDNP NMR screening experiment. The ligands are polarized through the radical pair mechanism with an excited photosensitizer (e.g., light excitation), and the NMR traces are recorded with a classic ^1^H 1D NMR experiment. (A) All of the ligands in the absence of a target yield a photo-CIDNP NMR trace that is maximal. (B) In the presence of the target, the interacting ligands (blue) yield a less intense, or quenched, photo-CIDNP NMR trace, while the non-interacting ligands (red) yield an identical photo-CIDNP NMR trace to the one obtained in the absence of the target.

As it is observed from eqs 2 and 3, the polarized ligand (L*) signal is proportional to the free ligand concentration (*L*_0_), which decreases upon binding to a protein due to steric hindrance, and is inversely proportional to the relaxation rate *R*_1,eff_. The combination of these two effects yields the quenching of the photo-CIDNP hyperpolarized signal in the case of binding to a protein, as *L* decreases and *R*_1,eff_ increases. Experimentally this translates into a reduction of the peak integrals and a hit can be detected by measuring the polarization ratio (eq 5)
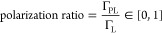
5With Γ_L_ and Γ_PL_, the peak integrals were obtained for two different samples: (1) in the absence of a protein and (2) in the presence of a protein, respectively, with a polarization ratio of 0 being the total binding of the ligand population to the target and 1 the absence of interaction. The principle of the photo-CIDNP ^1^H one-dimensional (1D) NMR screening experiment is shown in Figure 1.

From the Taylor expansion (eq 2), one can observe that in the initial regime, the polarization ratio (eq 6) depends only on the free ligand concentration in the presence and absence of a protein. The free ligand concentration *L* depends on the dissociation constant (*K*_d_), and the affinity can be found using the relation
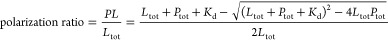
6where *L*_tot_ and *P*_tot_ are the total ligand and protein concentrations, respectively.

However, in the steady-state regime, the effect of the relaxation of the ligand starts and reduces the polarization ratio improving the contrast between the experiments with and without protein, i.e., the operative range. To achieve sufficient signal-to-noise at low micromolar concentrations, the photo-CIDNP experiments require light irradiation times of several hundreds of milliseconds. Therefore, the experiments in this manuscript were performed in the steady-state regime, which is reached for times greater than 1/(*R*_1,eff_ + *k*_cidnp_), typically in the tens of milliseconds range (eq 4).

**Figure 2:**
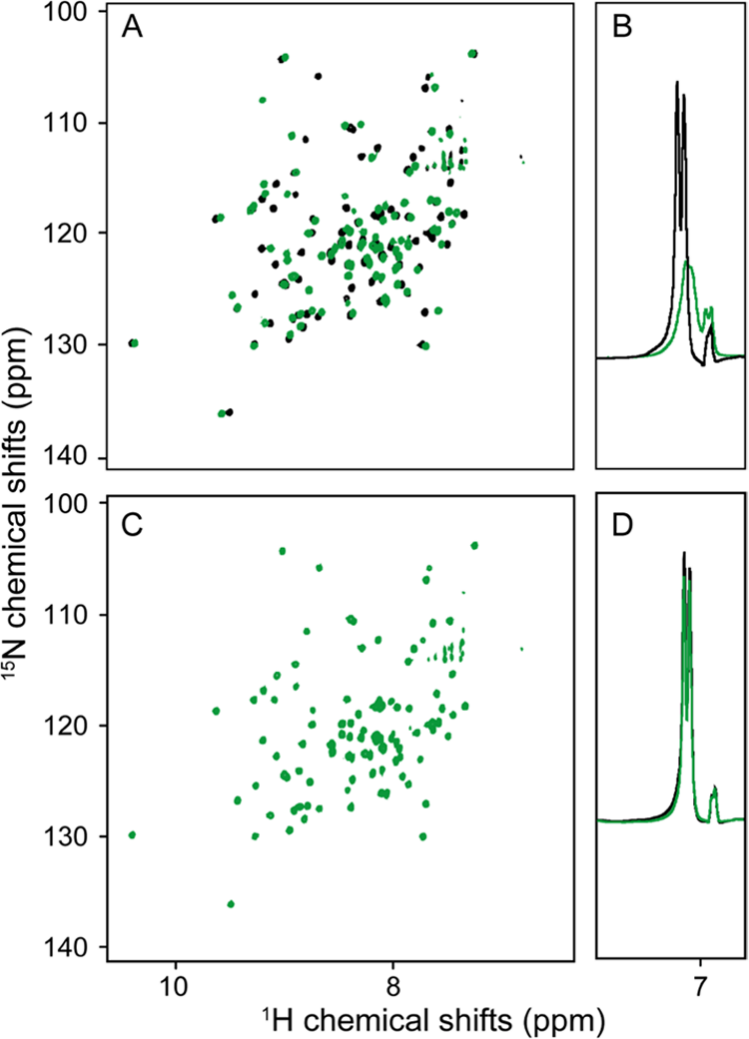
Peptide binding studies of peptides to the PDZ2 domain of the human tyrosine phosphatase by standard chemical shift perturbation 2D NMR experiments and photo-CIDNP 1D ^1^H NMR. (A) and (C) 2D [^15^N,^1^H]-HSQC spectra of the ^15^N-labeled PDZ2 domain in the absence (black) and presence (green) of the peptide WVSAV (A) or WEKLQT (C), the experiments were recorded each within ca. 30 min. Photo-CIDNP ^1^H 1D NMR spectra of the tryptophane aromatic resonances of (B) WVSAV and (D) WEKLQT in the absence (black lines) and presence (green lines) of the PDZ2 domain of the human tyrosine phosphatase, the experiments were recorded each within ca. 5 s. All of the 1D NMR spectra are single-scan experiments and were recorded with 100 μM peptide, ±100 μM PDZ2 domain at 298 K. The hyperpolarization was triggered by 4 s of light irradiation (450 nm, 1 W) in the presence of 25 μM fluorescein in photo-CIDNP buffer.

To establish a proof of concept, we decided to design and study peptides that bind to the PDZ2 domain of human tyrosine phosphatase comprising a tryptophane at the N-terminus of the peptides for detection by photo-CIDNP since tryptophane is known to be well polarized in the presence of fluorescein.^10,25^ The positive and negative control peptides (i.e., WVSAV and WEKLQT, respectively) were then checked in their binding capacity using [^15^N,^1^H]-HSQC experiments (Figure 2A,C). The WVSAV peptide-induced chemical shift perturbations (CSPs) indicative of binding can be observed in the spectra comparison (Figure 2A), while the lack of shift changes upon the addition of the peptide WEKLQT (Figure 2C) is attributed to the absence of binding. Furthermore, a [^15^N,^1^H]-HSQC-based titration series of the positive control peptide (WVSAV) revealed a *K*_d_ of 111 ± 3.3 μM calculated by Titan.^26^

The binding of WVSAV to the PDZ2 domain using photo-CIDNP 1D ^1^H NMR is demonstrated in Figure 2B by signal reduction of the polarized tryptophan resonances because the bound WVSAV is not polarized and its longitudinal relaxation (*R*_1,eff_) is increased, according to the theory introduced above. A polarization ratio of 0.35 is calculated (eq 6) for the WVSAV peptide upon the addition of the PDZ2 domain. The time dependence of the polarization ratio was investigated by varying the irradiation time in a range from 25 to 900 ms and was found to be constant over time (Figure S1), indicating the predominance of the steady-state regime after a few dozen milliseconds of irradiation (eq 3), suggesting an increase in the *R*_1,PL*_ of one to two log as compared to *R*_1,L*_. In addition, chemical shift changes of the polarized tryptophan resonances also indicate binding to the PDZ2 domain. The lack of binding to the PDZ2 domain of the peptide WEKLQT is evidenced by the lack of signal quenching of the polarized tryptophane resonances in the 1D ^1^H NMR spectrum (Figure 2D).

## Results

To establish photo-CIDNP-based small-molecule screening, the following aspects were studied: (i) exploration of the chemical space of the photo-CIDNP active small molecules, (ii) establishment of a photo-CIDNP compatible fragment library, and (iii) the screening of the library against the cancer-related protein PIN1. Upon establishment thereof, (iv) optimization in terms of small molecule and protein amounts required and (v) screening automation were investigated.

### Chemical Space Exploration

**Figure 3:**
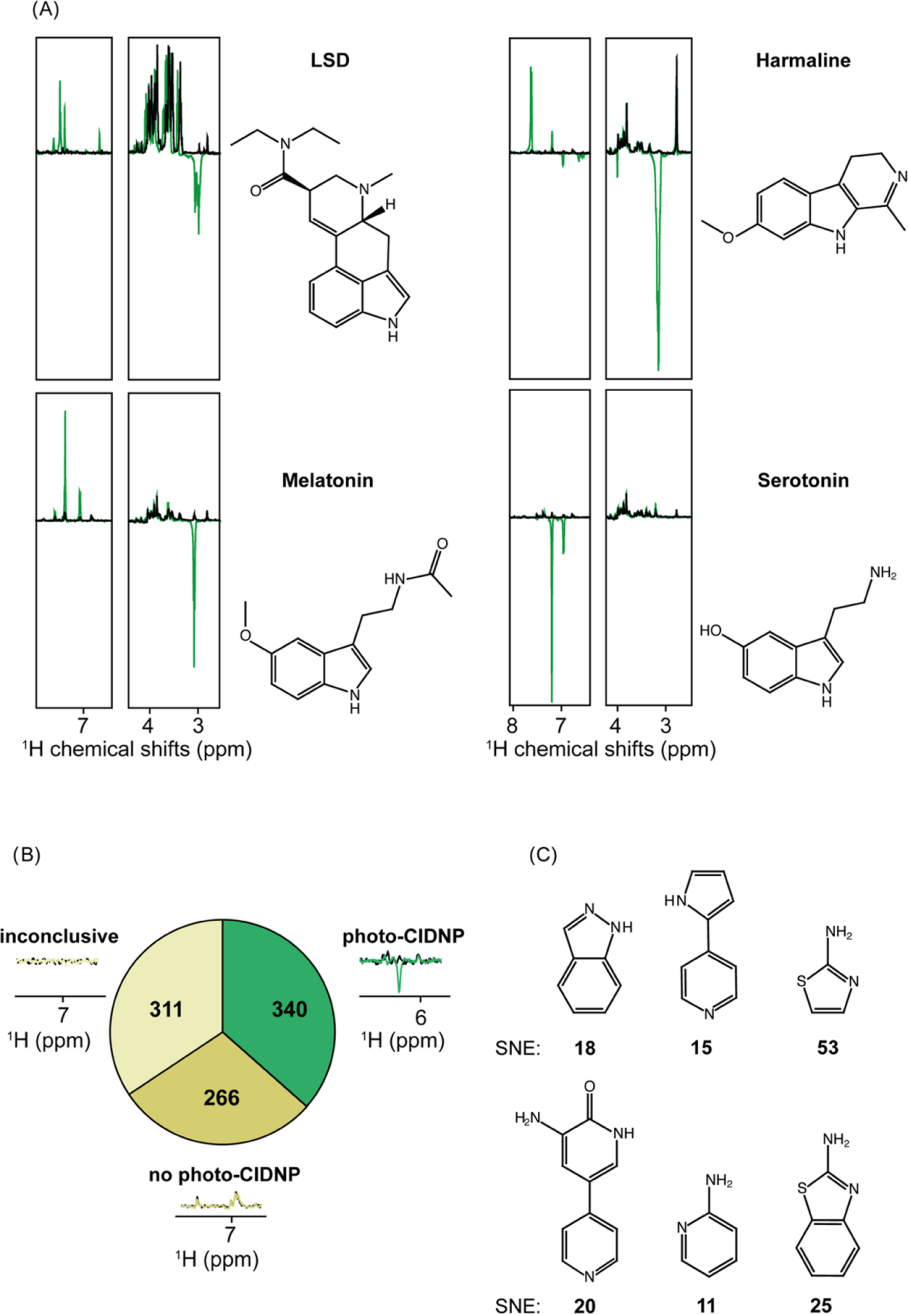
Exploration of the chemical space of small molecules compatible with photo-CIDNP hyperpolarization. (A) Photo-CIDNP ^1^H 1D NMR spectra of a series of indole-containing bioactive molecules, lysergic acid diethylamine (LSD), harmaline, melatonin, and serotonin. The black lines are the 1D spectra in the absence of light irradiation (single scan), and the green lines are the irradiated spectra. (B) Pie plot with the number of photo-CIDNP active compounds (green), including the spectrum of an example of positive photo-CIDNP spectrum in green; the number of compounds yielding no photo-CIDNP effect (kaki) along with an example of a spectrum; and the number of compounds for which no conclusion could be obtained (beige) with an example of the observed spectrum. The black spectra shown are the dark and the colored spectra correspond to the light-excited photo-CIDNP ^1^H 1D NMR spectra. (C) The example of new aromatic systems with different signal-to-noise enhancement (SNE) upon photo-CIDNP experiments. All of the molecules in (A), (B), and (C) were measured at 100 μM and the irradiated spectra were irradiated for 2 s at 450 nm (1 W) in the presence of fluorescein (25 μM), prior to recording the NMR spectra.

So far, only about 30 molecules have been reported to be suitable for photo-CIDNP nuclear polarization; among them, 7 were first described by us in a previous attempt to explore the chemical space of photo-CIDNP activity (Table S1).^13^ Amongst them are the amino acids tyrosine and tryptophan and their derivatives. Peptides comprising tryptophan or tyrosine, as demonstrated with the two peptides above, can be therefore used for ligand screening. Interestingly, it appears that the indole, phenol, and imidazole features found in tryptophan, tyrosine, and histidine, respectively, are present in an important number of bioactive molecules comprising FDA-approved drugs. We quantified the frequency of these features using the ChemBL database.^27^ We selected the molecules containing at least 1 aromatic ring and with a size below 300 Da, and we found that 11,485 molecules out of the pool of 225,196 molecules contained at least one indole, a phenol, or an imidazole ring, representing 5.1% of this specific chemical space. Regarding the deposited FDA-approved molecules in the ChemBL database, 648 of the 3340 molecules (19.4%) contained an aromatic ring which was an indole, a phenol, or an imidazole. We only considered the FDA-approved molecules containing at least one aromatic ring without any cutoff on the molecular weight. The reason for this overrepresentation of such features is likely to be evolutionary selection, although the question remains open and beyond the scope of this investigation. Nevertheless, bias in the bioactive chemical space is a hot topic in the medicinal chemistry field.^28,29^ Indole-containing drugs are particularly represented in psychoactive molecules such as lysergic acid diethylamide (LSD), harmaline, melatonin, and serotonin. Strong signal-to-noise enhancement (SNE) is observed for all of these molecules upon light irradiation in the presence of fluorescein (Figure 3A). The SNE is defined as the absolute difference between the signal integrals with and without irradiation normalized by the integral of the signal without prior light irradiation, , and Γ_light_ and Γ_dark_ are the signal integrals with and without prior light irradiation, respectively; the term dark spectrum refers to a spectrum recorded in the absence of prior light irradiation. The SNE reflects the relative molecule polarization obtained after a photo-CIDNP NMR experiment.

The presence of these aromatic rings in an important proportion of the bioactive chemical space suggests that if more aromatic rings are found to be compatible with photo-CIDNP hyperpolarization, an important part of the chemical space could be covered, opening the way to generalist approaches such as fragment-based drug design (FBDD).

The question arises whether the apparent scarce number of chemical features available for photo-CIDNP could be extended and thus enable the implementation of the technique for molecules other than peptides with tryptophane, tyrosine, or histidine tags. In an attempt to answer this question, the chemical space of photo-CIDNP active molecules was explored under the assumption that many other photo-CIDNP suitable molecules and chemical features exist and are simply unknown to this day. Therefore, a number of molecules with diverse aromatic scaffolds were selected from the EU-OPENSCREEN (EOS) collection. The primary objective was the identification of a novel aromatic scaffold, and the molecule properties, such as log *P*, molecular weight, or drug-likeliness, were disregarded. Finally, a selection of 917 molecules with different aromatic scaffolds and substitutions (i.e., hydroxy groups, amino, sulfamine, etc.) was screened under standard photo-CIDNP conditions in the presence of fluorescein (see Materials and Methods for more details). To extend the dataset, 468 molecules from the lab stock were included in the study. The EOS compounds were screened by us at the Latvian Institute of Organic Synthesis, an EOS Medicinal chemistry site using the same laser diode as used in Zurich at ETH because it could be installed in an hour. This demonstrates the agility of the photo-CIDNP setup and is another argument pleading in favor of the photo-CIDNP technique in NMR-based small-molecule screening.

The data was analyzed by visually comparing the dark and irradiated spectra (0 versus 2 s light irradiation). Whenever a compound exhibited a significant difference in the signal intensities (anomalous lines), the compound was classified as photo-CIDNP “active” (Figure 3B). The compounds showing no difference between the dark and the irradiated spectra were disregarded. The absence of anomalous lines has two causes: (1) the compound does not react with the fluorescein to form a radical pair and is therefore “inactive” and (2) the compound is not present in the sample due to solubility issues or the absence of compound in the stock solution, and the data is therefore inconclusive. The results of this chemical space exploration are summarized in Figure 3B. From the EOS exploration, the signal enhancement could be observed for 340 molecules, the absence of photo-CIDNP activity was observed for 266 compounds, and 311 compounds were inconclusive due to the absence of signal in the dark state attributed to poor solubility. We exclude the inconclusive molecules from our analysis and estimate that 56% of the selected molecules are hyperpolarizable using fluorescein-based photo-CIDNP. Selected examples of new aromatic rings compatible with photo-CIDNP are shown with their respective SNEs in Figure 3C. One should note that the SNE will vary depending on the utilized photosensitizer due to the difference in the *g*-factors of their radicals. Choosing a different photosensitizer has proven to be an efficient strategy for optimizing the SNE of a given molecule.^10,12,13^ From the 468 molecules screened in-house, 153 were photo-CIDNP active (SNE > 5) and 315 were not active (SNE < 5), yielding a polarizable rate of 33%. These findings provided the starting points to generate a fragment screening library with sufficient chemical diversity.

### Library Design

To design a small-molecule library for fragment-based photo-CIDNP NMR screenings, the selection of molecules comprising aromatic features with a strong photo-CIDNP-induced SNE identified from the chemical exploration discussed above was used to generate a subspace of the Chemspace catalogue. This subspace was refined in Datawarrior^30^ using filters according to the rule of three, such as molecular weight < 300 Da, log *P* < 3, or H-donors < 3.^31^ In addition, we applied a drug-likeliness filter with a cutoff value > −2.0.^30^ To reduce the library further, we clustered the library and generated for each cluster the most representative molecule to create a diversity set. In doing so, we designed a library of 287 compounds containing 104 different Murcko’s scaffolds^32^ (1 scaffold for 3 molecules on average) and 71 most central ring systems (1 ring system for 4 molecules on average).^33^ In comparison, the DSI-poised library, a reference for the FBDD field, contains 96 most central ring systems for 896 fragments in total, or on average 1 ring system for 11 molecules.^34^ Nevertheless, the DSI-poised library contains 447 different Murcko’s scaffolds, which is of higher diversity than our library (1 scaffold for 2 molecules on average). The reason for this is that Murcko’s framework accounts for the different ring systems of the same molecule connected together as one scaffold.^32^ Nevertheless, the connection of different cycles, aromatic or not, does not affect the photo-CIDNP SNE, and in our continuous effort to increase the library size, such a strategy will be implemented to improve the diversity of the fragment selection. The selection of fragments described here constitutes the pilot version of photo-CIDNP-compliant fragment libraries named NMhare. The presented version is named NMhare1.0 for being the first version of the library.

**Figure 4:**
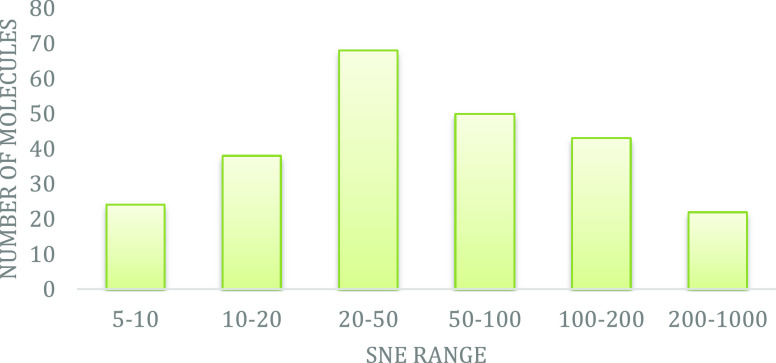
Distribution of the photo-CIDNP-based signal-to-noise enhancement (SNE) in the molecule library NMhare1.0. The SNE is obtained by normalizing the signal integrals of the irradiated spectra over the corresponding signal integrals of the non-irradiated spectra. As the intensity of the photo-CIDNP hyperpolarization varies due to the different hyperfine couplings of the individual nuclei,^9,21,24^ we use for ranking the strongest SNE observed per molecule for the ranking.

The library was subjected to quality control (QC) to verify the correct identity of the compound purchased, monitor impurities, and ensure the compound’s solubility in the aqueous buffer; this was done by recording 1D ^1^H NMR experiments of each compound at 200 μM in the presence of 22 μM DSS. The QC results are summarized in Table 1 and Figure 4. A first round of exclusion was performed by verifying that chemical shifts observed by 1D NMR matched with the fragment structures (277, 97%). Most compounds (262, 91%) were soluble at least at 100 μM. The solubility was verified by comparing the signal integrals of the fragments with the reference signal integral (DSS, at 0 ppm). This high solubility percentage was expected as fragments are generally fairly soluble in the μM range, and we included fragments using a conservative value for the log *P* (<3). Finally, the photo-CIDNP hyperpolarization in the presence of fluorescein was verified for the soluble fragments using photo-CIDNP 1D ^1^H NMR. These experiments were recorded at 50 μM fragment concentration in photo-CIDNP buffer comprising 25 μM fluorescein and an oxygen-quenching enzymatic mixture (see Materials and Methods).

**Table 1:** Quality Control Process of the NMhare1.0 Library^a^

correct ^1^H NMR	soluble	SNE with photo-CIDNP NMR	total
277	262	212	287
97%	91%	74%	100%

a First 287 molecules are selected to have a most central aromatic ring yielding photo-CIDNP SNE. Second, a 1D ^1^H NMR experiment of each compound (200 μM) is recorded in the presence of 100 μM DSS as a reference signal, and the signal intensity is compared to the DSS to ensure that the compounds are present at a concentration of at least 100 μM. Finally, the photo-CIDNP signal is recorded, and observation of SNE > 5 is the final criterion for inclusion.

We observed photo-CIDNP-induced SNE for 81% of the small molecules, i.e., we observed an SNE superior to 5-fold enhancement for 212 fragments of the 262 that we tested. This high percentage of photo-CIDNP activity validates our approach of exploring and including new aromatic moieties to select the library for photo-CIDNP compatibility. In summary, 74% of the molecules that were ordered passed the QC and were included for further screening. The SNE distribution was quantified for the fragments, of which 9% were above SNE 200, 18% between SNE 100–200, 20% between SNE 50–100, 28% between SNE 20–50, 16% between SNE 10–20, and 10% below SNE 10 (Figure 4). An SNE of 20 or more is important to access low-concentration screening applications. Nevertheless, we demonstrated in previous work that using different photosensitizers is an efficient approach to improving the SNE for a given molecule.^12,13,35^

### Photo-CIDNP Small-Molecule Screening for Ligand Binding to Human PIN1

To demonstrate the power of the single-scan photo-CIDNP-based NMR screening, the NMhare1.0 fragment library was screened against PIN1. PIN1 is a proline cis-trans isomerase whose biological significance includes a protective function against Alzheimer’s disease,^14^ an involvement in the regulation of mitosis (Lu et al., 1996),^15^ and an increase in hepatitis C infection.^17^ It is also overexpressed in many human cancer cells.^16^ It is thus regarded as a potential drug target, and several studies attempted to discover hits and unravel the structure–activity relationship.^3,4,36^ The screening conditions were 50 μM ligand in the absence and presence of 25 μM PIN1 in 20 mM phosphate and 50 mM sodium chloride comprising photo-CIDNP relevant constituents (i.e., 400 nM glucose oxidase, 200 nM catalase, 5 mM d-glucose, 20 μM fluorescein^10,37^). The NMR experiments were performed using single-scan NMR experiments, comprising light irradiation, an excitation pulse, and a water suppression scheme, as described previously.^13^ Although each experiment was recorded in only 2 s, the limiting step was the sample tube exchange. Indeed, the optic fiber needed to be plugged into the tubes while changing the samples, which required ca. 15 s per tube using a house-made connector tightening the tube and the optic fiber with O-rings (Figure S2). Removing the sample from and bringing the new sample into the spectrometer requires an additional 30 s, and a shimming step must be performed for each new tube (30 s). In total, the screening of 212 fragments with this rather primitive setup required 424 samples and roughly 11 h of experiment time.

As proposed in the theoretical part, the photo-CIDNP traces were recorded in the presence and absence of PIN1. The peak intensity and chemical shifts were analyzed pairwise to identify the hits. The visual inspection of the data identified 32 hits (15.1% hit rate), using as criteria a significant reduction of the polarized traces (20%, i.e., polarization ratio <0.8, which is obtained as described in Theory). Exemplary spectra of non-binders, together with their polarization ratios, are provided in Figure S3. The inspection of the NMR spectra that were recorded without prior light irradiation (dark spectra) led to the exclusion of 12 molecules for which the polarization ratio was below 0.8. Indeed, different ligand concentrations in the dark spectra render the analysis inconclusive, forcing us to discard these hits and consider them as false positives. Such concentration differences can be in part attributed to pipetting errors during the sample preparation, which was performed manually for the 424 samples (please note, in order to improve the robustness of the sample preparation, we recommend the use of a liquid handling robot). In return, the possibility of studying the dark spectrum enables the elucidation of pipetting errors straightforwardly. The 20 remaining hit fragments were validated by recording [^15^N,^1^H]-HSQC NMR spectra of ^15^N-labeled PIN1 (50 μM) at a fragment concentration of 200 μM, which was compared to a reference apo-PIN1 [^15^N,^1^H]-HSQC NMR spectrum to identify chemical shift perturbations. The reference apo-PIN1 sample was spiked with 1.9 μL of DMSO to compensate for the DMSO that is introduced upon fragment addition, as the fragments are stored in stock solutions of 5 mM 90% DMSO-*d*_6_ and 10% D_2_O. Fragment-induced CSPs were observed for all of the 20 primary hits from the photo-CIDNP screening (Table S2). Figure S4 shows the structures of the different hits, and Table S2 reports the polarization ratios (eq 6) and the average CSPs observed in the [^15^N,^1^H]-HSQC. Fragments **1** and **13** are known inhibitors of PIN1^3,4^ and exhibit strong CSPs located at the known binding site of PIN1, which is also the catalytic site for the cis/trans-proline isomerization. The fragments **10**–**12** show some structural proximity with juglone, a reference inhibitor of PIN1,^38^ and **10** and **11** show significant CSPs at the catalytic site of PIN1 (Figure S5). Another compound that exhibited interesting CSPs at the catalytic site of PIN1 is **3**, which contains an indazole ring similar to the indole ring of compound **1** (Figures S4 and S5). However, compound **3** does not show any of the key features of **1**, such as the carboxylate moiety and the methyl in the benzyl part, which fits in a hydrophobic cliff in the catalytic pocket of PIN1 and is known to contribute to forming van der Waals interactions for both compounds **1** and **13** (residues L122, M130, F134, and T152).^3,4,36^ Overall, the CSPs induced by the 20 fragments were all located at the catalytic site, and many of these CSPs were co-observed for known inhibitors **1** and **13** as well as for the other fragments (Figure S5). Interestingly, a class of hits (compounds **15** to **19** in Figure S4) exhibited more or less severe deterioration of the PIN1 secondary structure, observed by [^15^N,^1^H]-HSQC (Figure S6). The reason for that is hypothetical and beyond the scope of this study.

**Figure 5:**
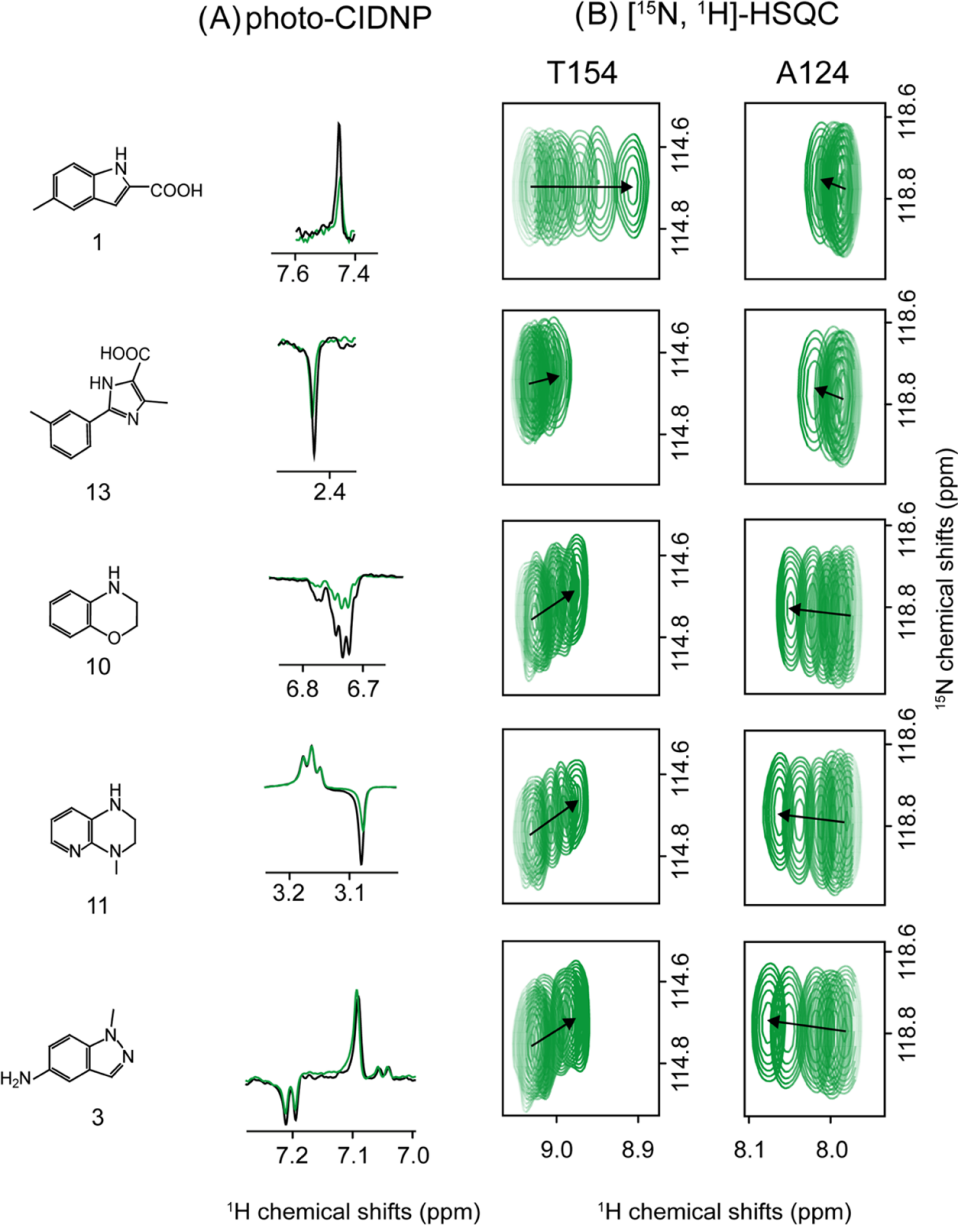
Selected validated hits from screening of Nmhare1.0 against PIN1 using photo-CIDNP ^1^H 1D NMR. (A) Signal quenching of different fragments upon binding to PIN1 (compounds **1**, **13**, **10**, **11**, and **3**) at fragment concentrations of 50 μM and PIN1 concentrations of 25 μM. (B) Chemical shift perturbation titration studies of the compounds with ^15^N-labeled PIN1 measured by [^15^N,^1^H]-HSQC experiments and shown for residues T154 and A124 in the presence of increasing ligand concentrations. Compound **1** was titrated at 0, 80, 100, 200, 300, 600, and 1000 μM compound concentrations; compound **13** titration series ranged over concentrations of 0, 80, 100, 200, 300, 600, 1000, and 2000 μM; compounds **3**, **10**, and **11** were studied at concentrations of 0, 200, 500, 1000, 2000, 3000 μM and all of the samples contained 80 μM of PIN1 protein and were measured in PIN1 buffer at 298 K.

The interactions of compounds **1**, **3**, **10**, **11**, and **13** were quantified by calculating the polarization ratio (Table S2) and by performing [^15^N,^1^H]-HSQC-based titration series with increasing ligand concentration. Figure 5 shows the relative photo-CIDNP polarized signal decay related to the presence of PIN1, which, according to the theory described above, can be attributed to target binding, together with the [^15^N,^1^H]-HSQC spectra used for hit validation.

The titration series with [^15^N,^1^H]-HSQC experiments for each fragment **1**, **3**, **10**, **11**, and **13** are shown in Figure 5 as well, and the resulting dissociation constants (*K*_d_) were derived from fitting the CSP build-up curves (Figure S7) and are provided in Table 2. For both **1** and **13**, the known ligands of PIN1, *K*_d_s of 1.5 and 1.7 mM, were obtained. It is noted that these values contrast with our own literature, which is explained by the fact that for fragment **1**, the affinity had previously been measured on a different construct (R14A/Q131 mutant) and at different pH values,^3^ and for fragment **2**, the *K*_d_ had been obtained indirectly.^36^ The *K*_d_s obtained for the other fragments, **3**, **10**, and **11**, were higher than the maximal fragment concentration in the titration series and therefore should be considered semi-quantitative. Nevertheless, these data consolidate photo-CIDNP-based fragment screening as an approach that can detect weak protein–ligand interactions up to the millimolar range. Furthermore, no false positive binders were detected in the primary screening, which facilitated the hit validation process. The compounds **1**, **3**, **10**, **11**, and **13** have polarization ratios of 0.42, 0.75, 0.38, 0.53, and 0.38, respectively, which do not necessarily correlate with the *K*_d_s of 1.5, 12, 8.4, 5.4, and 1.7 mM, respectively (Table 2). This is attributed to the fact that in the steady-state regime, the selective relaxation rate plays an important role in the value of the polarization ratio. The selective relaxation rate of the bound polarized ligand, including the auto-relaxation term, depends as well on the ligand pose.^39^ For this reason, the quantitation of the polarization ratio with regard to the ligand affinity should be considered with caution.

**Table 2:** Dissociation Constants (*K*_d_) Measured by Titration Series of [^15^N,^1^H]-HSQC with Increasing Ligand Concentrations and 200 μM PIN1^a^

compound	*K*_d_ T154 (mM)	*K*_d_ A124 (mM)	polarization ratio	*T*_1ρ_ decay ratio	STD
**1**	1.5	3.6	0.42	0.67	+
**13**	1.7	4.5	0.38	0.45	+
**10**	8.4*	11.8*	0.38	0.90	–
**11**	5.4*	8.8*	0.53	0.92	–
**3**	12*	8.6*	0.75	0.98	–

a The *K*_d_s were obtained from fitting with eq 6 in the CcpNMR software 3.0.^40^ Fitting curves are provided in Figure S7. The values annotated with * could not be fitted to a maximum and therefore should be considered with caution. The proposed *K*_d_s are most likely to be higher than these values. The polarization ratios were calculated according to eq 6 and showed a trend with low values for higher affinities, with the exception of compound **10**, which shows a low polarization ratio despite a low affinity. The *T*_1ρ_ decay ratio and the saturation transfer difference (STD) were measured for samples containing 200 μM ligand (*L*) and 200 μM ligand with 20 μM protein (PL). The *T*_1ρ_ decay ratio was calculated with the formula (Γ_400ms_/Γ_10ms_)_PL_/(Γ_400ms_/Γ_10ms_)_L_. The observation of ligand signals in the STD NMR spectra detects a protein–ligand interaction; (+) the absence of a signal fails to detect protein–ligand interaction (−). Figure S8 provides examples of spectra for high (compound **1**) and low (compound **11**) *T*_1ρ_ decay ratios and the positive STD spectrum for compound **1**.

For comparison, the signal decay during a *T*_1ρ_ NMR experiment was measured at the same sample conditions as the photo-CIDNP experiments (Table 2). The signal decay between two *T*_1ρ_ relaxation delays of 10 and 400 ms is measured in the absence and presence of the PIN1 protein. The increase in signal decay is calculated as the ratio of the integral’s ratio, i.e., (Γ_400ms_/Γ_10ms_)_PL_/(Γ_400ms_/Γ_10ms_)_L_. The most affine binders, compounds **1** and **13**, show a significant reduction of the *T*_1ρ_ decay ratio upon PIN1 addition, witnessing an increase in the relaxation decay. The weaker binders, compounds **10** and **11**, show less intense yet observable decay acceleration upon protein binding, and compound **3**, which is the weakest binder according to the [^15^N,^1^H]-HSQC titration, shows the weakest difference decay. Interestingly, the 20% cutoff would discard compounds **10** and **11** if only the *T*_1ρ_ relaxation experiments were considered. The signal-to-noise of the *T*_1ρ_ spectra were ca. 300 for a number of transients of 512, corresponding to a measurement time of 25 min, while the photo-CIDNP experiments provided a signal-to-noise of ca. 100 for a single-scan experiment corresponding to a two second’s experiment. The STD experiment only shows a signal for compounds **1** and **13**, and a baseline perturbation is observed for compound **10** but not enough to qualify it unambiguously to the interaction with PIN1. The signal-to-noise of the STD NMR experiments^1^ was only 25 and 19 for compounds **1** and **13**, respectively, after 50 min measurement corresponding to a 512 transient’s experiment. This suggests that the reason why the compounds with high millimolar affinity could not be detected with this technique is due to the poor sensitivity of STD NMR.

### Photo-CIDNP Small-Molecule Screening at Low μM Fragment Concentration and in Pooled Fragment Samples

One of the bottlenecks of NMR-based screening is the requirement for a high amount of ligand and protein concentrations, which requests, on the one hand, large quantities of valuable entities and, on the other hand, limits the chemical space of available fragment molecules severely due to solubility issues. The high screening concentrations may also yield false positives in STD-based screening experiments attributed to micelle formation or aggregation of ligands.^41^ Using photo-CIDNP NMR, we can screen at low μM concentrations and thereby overcome the disadvantages of state-of-the-art NMR-based screenings.

**Figure 6:**
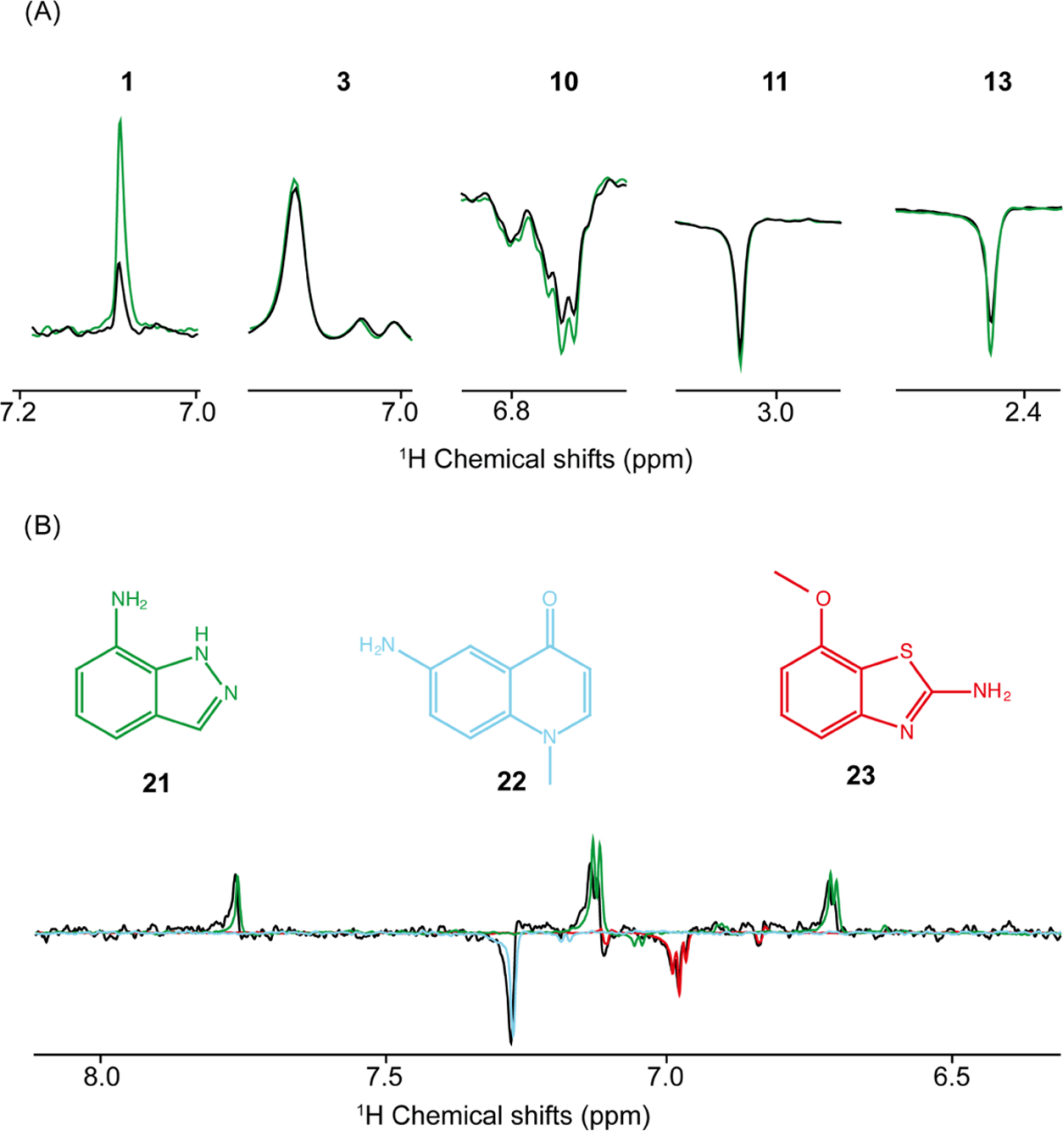
Photo-CIDNP fragment screening optimization by (A) lowering the concentration of protein and fragments and (B) by pooling fragments. (A) 1D ^1^H NMR spectra of compound **1** (5 μM), **3** (20 μM), **10** (20 μM), **11** (20 μM), and **13** (20 μM) in the presence (black) and absence (green) of PIN1 (2 μM). (B) photo-CIDNP NMR 1D ^1^H NMR spectra of the compounds **21** (green), **22** (cyan), and **23** (red) individually, and all three compounds pooled together (black). The samples with single compounds were prepared at 25 μM, and the pooled sample with 10 μM of each compound. All of the spectra are single-scan experiments after 2 s light irradiation (450 nm, 1 W). The signal intensities of the individual spectra were adjusted individually to the black spectrum for the sake of visualization.

In an attempt to estimate how low the concentration for screening could be using our approach, photo-CIDNP 1D ^1^H NMR experiments of compounds **3**, **10**, **11**, and **13**, in the absence and presence of PIN1 at 20 μM ligand concentration and 2 μM protein concentration were measured. Encouraged by the strong SNE of compound **1**, this fragment was additionally measured at 5 μM in the absence and presence of 2 μM PIN1. As demonstrated in Figure 6A, clear photo-CIDNP quenching is observed in a single-scan 1D ^1^H NMR experiment at the low micromolar concentration for compounds **1**, **10**, and **13** with the polarization ratios of 0.45, 0.76, and 0.73, respectively. Compounds **3** and **11** do not show clear quenching of the photo-CIDNP effect (Figure 6A), although a trend can be noted with polarization ratios of 0.95 and 0.86, respectively. We attribute this absence of significative quenching to the very weak affinities of these molecules of >12 mM for compound **3** and >8.8 mM for compound **11**, respectively. By acquiring multiple scan experiments, the required screening concentrations are likely to be further reduced. For comparison, the *T*_1ρ_ relaxation experiments performed with the same sample conditions could detect a difference in the signal decay rates upon protein addition only for compounds **1** and **13**, with *T*_1ρ_ decay ratios of 0.80 and 0.76, respectively. The rest of the compounds (**3**, **10**, and **11**) provided a decay ratio of 1.0. Furthermore, the signal-to-noise of the *T*_1ρ_ relaxation experiments ranged from 14 to 85 for the highly scalarly coupled aromatic protons of compound **10** to the methyl peak of compound **13**. This compares with the signal-to-noise values of photo-CIDNP NMR experiments at a concentration of 20 μM, which were 255, 81, 25, 234, and 75 for the compounds **1**, **3**, **10**, **11**, and **13**, respectively. The *T*_1ρ_ relaxation NMR experiments took 25 min (512 transients) to record, while the photo-CIDNP experiments took only 2 s. The STD NMR experiment was unable to detect the interaction of any compounds with the target.

To further reduce the consumption of protein in the photo-CIDNP-based screening, fragments were pooled into one NMR sample, as often done in standard NMR-based screening experiments.^42^Figure 6B shows the photo-CIDNP enhanced 1D ^1^H NMR spectra of a pool of compounds **21**, **22**, and **23** in the absence of PIN1, demonstrating the possibility of pooling compounds to save time and target consumption. In the case of ^1^H-based photo-CIDNP, as presented here, pooling is limited to 3 to 4 compounds because, for 1D ^1^H NMR spectra, extensive chemical shift analysis is necessary to avoid peak overlap due to the limited chemical shift range and multiple peaks per compound. This could be overcome by using ^19^F-based photo-CIDNP experiments (see Discussion).

### High Throughput and Automation

**Figure 7:**
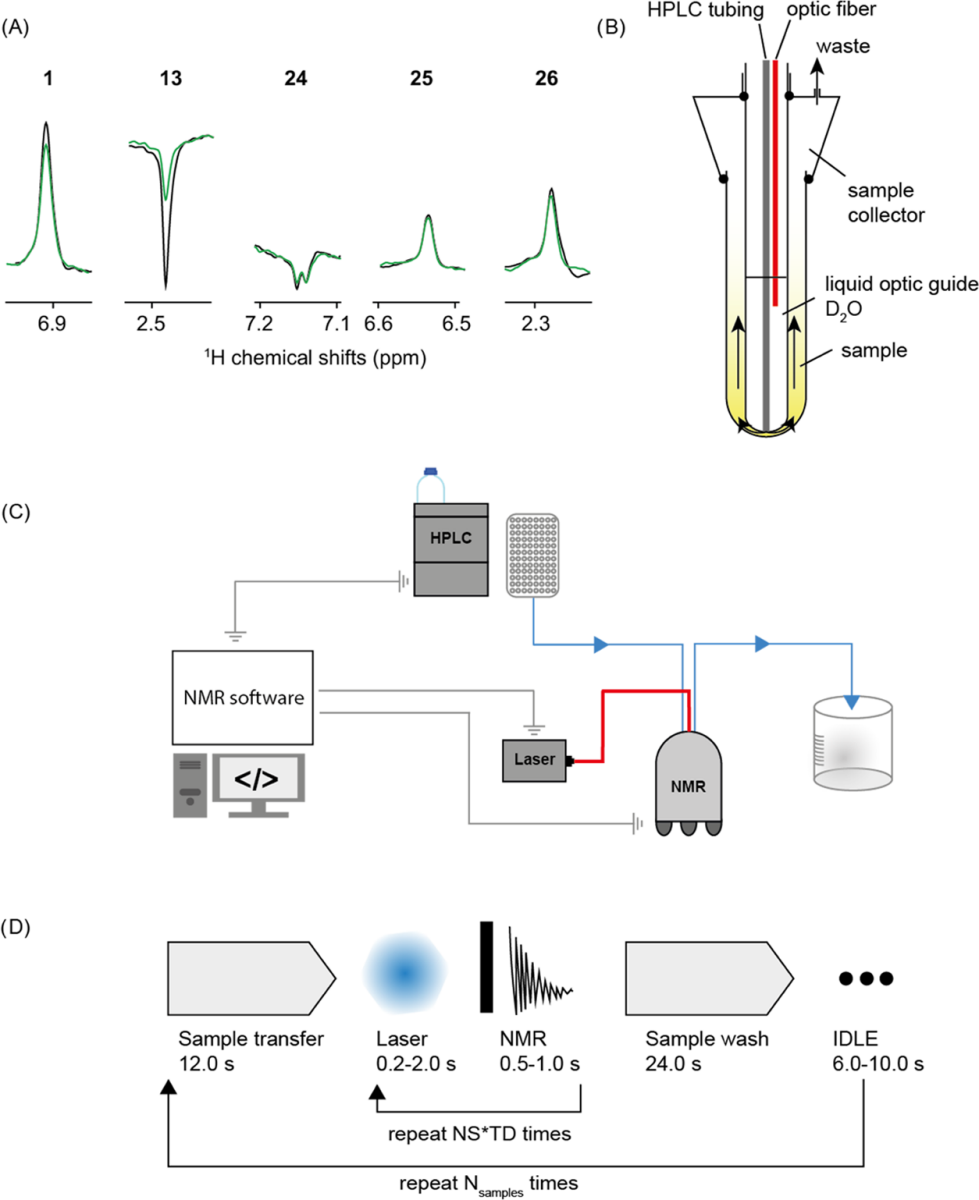
Automated screening of PIN1 with Nmhare1.0 library using photo-CIDNP 1D ^1^H NMR. (A) Automated Photo-CIDNP 1D ^1^H NMR fragment screening using a flow cell system applied to PIN1. 1D ^1^H NMR spectra of compounds **1**, **13**, **24**, **25**, and **26** in the absence (black) and presence (green) of the PIN1 protein. As described above, **1** and **13** are known binders of PIN1, while **24**, **25**, and **26** were not identified as binders. The samples were prepared with 50 μM of compounds and 20 μM of PIN1 protein in PIN1-photo-CIDNP buffer (see Materials and Methods). The samples were irradiated for 1.5 seconds at a power of 1 W (450 nm). (B) Schematic representation of the flow cell consisting of two coaxial tubes. The sample is injected at the bottom of the tube by the mean of a PEEK tubing and then is pushed into the wall, i.e., the space between the inner and the outer tube. (C) The HPLC and the NMR spectrometer are interfaced with a computer through TTL connections enabling communication between the two instruments. The laser is triggered using the Topspin software, and the TTL connection is made between the NMR console and the laser. (D) Workflow of flow-through photo-CIDNP NMR, the sample is injected by the autosampler of the HPLC and transferred in a fluidic connection to the flow cell located inside the NMR spectrometer (B). When the sample reaches the detection region of the NMR spectrometer, the flow is stopped by switching the flow path by the mean of the valve. The photo-CIDNP NMR experiment is then recorded with the pulse program triggering light irradiation, and subsequently performing the NMR experiment acquisition, the NMR experiment can comprehend different numbers of transients (NS) and indirect time dimension points (TD) that will increase the measurement time accordingly; finally, the sample is washed by restoring the flow in the fluidic connection. The operation is repeated for each sample.

While several other hyperpolarization methods yield higher SNE and have been established for ligand binding detection, their implementation into a highly automated system remains challenging.^43,44^ Photo-CIDNP hyperpolarization presents the advantage of being generated *in situ*, at room temperature, in solution, simply by shining light on the sample. To illustrate the agility of the photo-CIDNP ligand screening approach, we designed a flow-through setup compatible with a cryoprobe (Figure 7C). The setup consists of a pump, an autosampler, a valve, and a 3D printed flow cell (Figure 7B,C), which comprises an optic fiber for light irradiation coming from a 1.6 W diode laser emitting at 450 nm from Thorlabs. The experiment is performed as follows: (i) the sample is injected, (ii) transferred from the autosampler to the flow cell thanks to the pump, (iii) once the sample has arrived at the flow cell, the flow is stopped by deviating the flow path toward the waste with the valve. (iv) While the flow is stopped, a photo-CIDNP ^1^H 1D NMR spectrum is recorded, as described above. Finally, (v) the flow path is restarted to wash the sample with buffer (Figure 7D). For method robustness purposes, we chose to use a high-performance liquid chromatography (HPLC) pump, as they have good flow accuracy and can resist the important back pressure (100 bars for 2.5 mL/min) induced by the long transfer line (5 m). We tested our setup by rescreening 48 compounds, including the true binders **1** and **13**, against PIN1 within 96 min. As in the previous screening performed in NMR tubes, protein interactions of compounds **1** and **13** through the signal reduction in the presence of the PIN1 protein were observed (Figure 7A). The robustness of the system can also be checked by looking at the true negative compounds such as **24**, **25**, or **26**, for which the photo-CIDNP ^1^H 1D NMR spectra in the absence and presence of PIN1 show very similar intensities (Figure 7A). As expected, the photo-CIDNP 1D ^1^H NMR spectra are very reproducible, validating the accuracy of the sample transfer from the autosampler to the flow cell. Moreover, no sample contamination by residual compounds was observed from the previous sample attributed to the washing step of ca. 30 s.

The injection turnaround time of the sample is performed in 39 s, and ca. 10 s of IDLE time need to be added for method equilibration between the different methods (injection-transfer-stop flow-wash). Therefore, 48 molecules were screened in 96 min, corresponding to a screening of 1440 samples per day.

The autosampler that we used can contain 3 plates of 96 or 384 wells. Given that the injection volume is 100 μL, 384 well plates with working volumes of 120 μL could, in principle, be used. In modern HPLC systems, plate rack systems with capacities of up to a dozen of plates could be used for automation of the screening to a great extent. Some limitations of our current systems should be noted, giving room for improvement and further development. First, the flow cell contains a volume of ca. half of a conventional 3 mm tube, and the sample is located at the edge of the cell where the filling factor is less optimal; for these reasons, we observe a loss of the signal-to-noise of ca. 4-folds with a flow cell compared to the conventional 3 mm NMR tube (Figure S9). Second, sample irradiation remains challenging. Recent development using etched tubes conducting light for optimal sample irradiation is a promising opening to design a flow cell with improved light coupling.^45^ Third and final, the HPLC system that was refurbished into an autosampler system is 20 years old and cannot perform sample transfer at a rate faster than a sample per min. In principle, this rate could reach a sample per 6 s with the fastest HPLC autosamplers, corresponding to a rate of 14,400 samples per day. More realistically, an injection cycle of 20 s, comprising washing steps and NMR measurement time, which would correspond to a screening rate of ca. 5000 samples per day, seems feasible.

## Discussion

While NMR is a major method for fragment-based drug design, attributed amongst others to its minimal assay development, its probe to be the molecule’s atoms nuclei, and its ability to detect weak interactions, it suffers from low sensitivity. This is due to the small Boltzmann population difference between nuclear spin states at room temperature requiring long measurement times and high sample concentrations. Considerable efforts in sensitivity enhancement include dynamic nuclear polarization (DNP),^6^ para-hydrogen-induced polarization (PHIP),^7^ signal amplification by reversible exchange (SABRE),^8^ and chemically induced dynamic nuclear polarization (CIDNP).^18,19^ Applications to small-molecule screening with DNP,^44^ PHIP,^46^ and SABRE^43^ were presented. While some of the methods reach polarization enhancements of 10^3^ to 10^4^-fold, photo-CIDNP enhancements for ^1^H nuclei are typically only one to two orders of magnitude (Figure 4).^12,35^ Despite this (so far) moderate enhancement photo-CIDNP-based ligand screening, as presented here, is of interest here because it can be induced with the simple light excitation of a photosensitizer present in the aqueous sample at room temperature. The polarization of a small molecule is directly affected by protein binding (eq 6), allowing a straightforward high-sensitivity small-molecule binding diagnosis covering binding affinities with μM to mM *K*_d_ as presented (Figures 2 and 5, Table 2). In comparison to state-of-the-art 1D NMR experiments, photo-CIDNP NMR proves to be more sensitive to weak binders’ detection, which is attributed to the contribution of the auto-relaxation rate to the polarization ratio. Moreover, at low concentrations, single transient experiments (2 s) yield higher signal-to-noise than 30 min experiments. The measurement time is shown to be as short as a couple of seconds, yielding a theoretical screening rate of >10,000 samples per day. In practice, this experimental time is limited by the sample exchange and the shimming for each sample tube. Alternatively, a photo-CIDNP system without a glass fiber but using mirrors to deliver light from a laser or the use of LEDs^25,47^ can be constructed within a (commercially available) sample changer (such as the SampleJet from Bruker) and a standard NMR glass tube per measurement/sample and by implementing automated shimming solutions such as recently presented by Becker et al.^48^ One could then consider a screening throughput of 2000 samples per day and, with the pooling of several fragments, could screen up to 10,000 molecules a day. This number could be pushed further by several folds if ^19^F photo-CIDNP NMR experiments^47,49^ were implemented since there are ^19^F pooled libraries with 10–30 compounds, also reducing target consumption.^42^

In the meantime, by screening manually, we performed at a rate of ca. 1000 samples per day (424 samples in 11 h), which is 10 to 20-fold faster than the state-of-the-art ligand observed NMR screening. We also propose a simple and relatively low-cost setup to automatically screen samples at a rate of 1500 samples per day (Figure 7B,C), which could be readily improved with off-the-shelf hardware solutions to achieve a screening rate of ca. 5000 samples per day. Furthermore, the screening can be done at low μM ligand concentration, which is important as many larger fragments and drug-like molecules comprise a low water solubility, and a low μM protein concentration limits the protein consumption and herewith the costs per sample considerably. Prior work combining flow-through NMR and hyperpolarization was done, in particular in the field of dissolution DNP.^50^ As the polarization is performed *ex situ*, the sample needs to be swiftly transferred, involving a hot stream to thaw the ligand sample and inject it into a preloaded protein aliquot. The protein needs to be preloaded independently from the polarized ligand to maintain its structural integrity. To improve the throughput by a factor of two, the flow cell can be parallelized, with one path containing the ligand alone and the other the protein–ligand mixture.^51^ However, the flow-through DNP setup requires higher instrumentation than photo-CIDNP flow-through platforms regarding costs and operational complexity. A recent and more scalable alternative is the bullet DNP technology pushing the frozen sample with high-pressured gas into the buffer located in the probe.^52^ In both approaches, the sample transfer and mixing are faster than photo-CIDNP flow-through, which operates with classical HPLC pumps. DNP is, in principle, able to hyperpolarize any molecule without chemical space restriction, and its polarization performances^6^ are greater than for photo-CIDNP, which may help to reduce even further the sample concentrations. However, the *ex-situ* hyperpolarization through DNP is typically taking longer (minutes) than photo-CIDNP hyperpolarization (seconds). Considering the simplicity of implementation and the operational throughput, photo-CIDNP proves to be a more affordable and scalable method.

The main potential limitation of photo-CIDNP is the restricted chemical space since only a subset of molecules can be polarized by photo-CIDNP. Before this work, only about 30 molecules were identified to be photo-CIDNP-active^11,13^ because most of the research therein was focused on the exploration of the physical mechanism behind photo-CIDNP.^9,10^ The presented exploration of a chemical library comprising 917 compounds (Figure 3) not only enlightened that ca. 57% of the screened molecules were photo-CIDNP-active, and thus many fragments appear to be photo-CIDNP-active but also built a basis for the establishment of a fragment library that can be screened by photo-CIDNP NMR. On this basis, we also estimate that about 25–30% of all biologically relevant small molecules should be photo-CIDNP active. This selectivity introduces a chemical bias of the fragment libraries that can be designed. Nevertheless, a recent analysis of the desirable chemical space for fragment screening reveals that functional diversity can overcome a limited scaffold diversity,^53^ and we know that functional diversification modulates but maintains photo-CIDNP activity.^13^ Furthermore, it is preferable to design libraries with strong pharmacophoric relevance in order to achieve the desired chemical space coverage. A recent algorithm developed by Bajusz et al. is able to design a very reduced library of 96 fragments addressing more than 90% of the known pharmacophores.^28^ Although the implementation of the former and later concepts will significantly improve the chemical space coverage of photo-CIDNP fragment libraries, a part of the chemical space will remain uncovered. To overcome this limitation, we propose to use competition experiments, where once a lead compound is identified with photo-CIDNP screening, competition experiments with any molecule can be performed within the same photo-CIDNP screening setup.

In addition to the small-molecule screening discussed, also peptide binding using photo-CIDNP with peptides comprising tryptophan, tyrosine, or the very highly photo-active amino acid derivative HOPI^12^ can be screened as demonstrated for the PDZ2 study in Figure 2. Finally, it is mentioned that on the biomolecule side, any system, including soluble proteins and RNA of any size, membrane proteins embedded in detergents or vesicles, and proteins or RNA complexes derived from any sources (including mammalian cells), can be studied since the detection is on the ligand side.

## Conclusions

A continuous wave photo-CIDNP-based NMR small-molecule screening approach, including apparatus design, binding affinity determination methodology, setup of a small library, and proof of concept screening application, is presented without false positives. It indicates that a comprehensive, versatile, and straightforward high-throughput NMR screening approach for the identification of fragment binding to biomolecules in solution without protein-specific adaptation and at both low μM ligand and both low μM unlabeled protein/biomolecule concentration is established with the potential for screening 10^4^ compounds per day.

## Materials and Methods

### Peptides and Small Molecules

The peptides WVSAV and WEKLQT were purchased from BACHEM.

Small molecules screened were purchased from Molport or provided by EOS for exploring photo-CIDNP activity. The stock solutions of the libraries were 50 mM in DMSO-*d*_6_ in a 96-well plate format. The measurements were performed in 100 mM PO_4_ at pH 7, 25 μM fluorescein, 200 nM glucose oxidase, 140 nM catalase, and 2.5 mM glucose.^10,37^

The NMhare1.0 fragments were purchased from Chemspace. The stock solutions of the library were 50 mM in DMSO-*d*_6_ or 5 mM in 90% DMSO-*d*_6_/10% D_2_O (v/v), respectively, in a 96-well plate format.

### Expression and Purification of PDZ2 Domain and PIN1

Expression in *Escherichia coli* cells (BL21 (DE3)) and purification via Ni-NTA chromatography of both the PDZ2 domain of human tyrosine phosphatase 1E (hPTP1E) comprising an N-terminal polyhistidine tag and peptidylprolyl cis/trans isomerase, NIMA-Interacting 1 (PIN1) also comprising an N-terminal polyhistidine tag were carried out according to previously reported procedures.^54^ The selected NMR buffer for the PDZ2 domain comprised 150 mM sodium chloride and 50 mM phosphate at pH 6.8 (PDZ2 buffer), while for PIN1, it comprised 50 mM NaCl and 20 mM KPO4 at pH 6.8 (PIN1 buffer). ^15^N-labeled PIN1 and PDZ2 domains were expressed and purified for the control experiments following standard procedures using a minimal medium with ^15^N-labeled ammonium sulfate described elsewhere.^54^ No stable isotope labeling was required for the small-molecule screening experiments.

### NMR Experiments

The photo-CIDNP NMR measurements were performed at 298 K on a Bruker Avance III HD 600 MHz spectrometer equipped with a cryoprobe. The laser used was a Thorlabs L450P1600MM, a diode laser emitting at 450 nm. The laser light was coupled (using appropriate coupling optics) into an optical fiber (Thorlabs, FG950UEC) of length 10 m and a diameter of 0.95 mm. The end of the fiber was inserted into the sample solution inside the 3 mm NMR glass tube to a depth of about 5 mm above the NMR coil region. The library QC and the control binding experiments for PIN1 were measured on a Bruker Avance III HD 600 MHz spectrometer equipped with a cryoprobe and SampleJet.

To prevent photosensitizer quenching, the enzyme cocktail glucose oxidase (GO, 120 kDa), catalase (CAT, 240 kDa), and d-glucose (G, 180 Da) were used at a concentration of 200 nM, 140 nM, and 2.5 mM, respectively.^10,37^ The stock solutions were 4.0 μM for Go and 4.0 μM for Cat, respectively, in 10 mM NaPO_4_ buffer and pH = 7.2. The glucose stock solution was 500 mM in D_2_O with 0.02% NaN_3_.

For the PDZ2 domain screening experiments, the samples were prepared in the presence of 25 μM fluorescein, 100 μM peptide, and 100 μM protein constructs.

For the PIN1 screening experiments, all of the samples were prepared in the presence of 20 μM fluorescein, 50 μM compound, and 25 μM protein constructs in PIN1 buffer.

For the research of new photo-CIDNP polarizable molecules, the samples were prepared using a Tecan Freedom EVO 100 pipetting robot in the presence of 25 μM fluorescein and 100 μM compound. The GO and CAT concentrations were identical as previously described, and the buffer was 100 mM KPO_4_ at pH = 7.2.

The QC for the NMhare1.0 library was performed at 200 μM compound concentration in 200 mM KPO_4_ at pH = 7.2 containing 22 μM DSS.

For the control binding experiments using ligand-induced chemical shift perturbation analysis, standard [^15^N, ^1^H]-HSQC spectra were measured. The detailed conditions for the 2D experiments acquired at 600 MHz ^1^H NMR frequency were the 100 μM ^15^N-labeled PDZ2 domain in the absence and presence of equimolar peptide in PDZ2 buffer and at a temperature of 298 K and 50 μM ^15^N-labeled PIN1 in the absence and presence of 200 μM ligands in PIN1 buffer and at a temperature of 298 K, respectively. Typically, 220 (*t*_1,max_ (^15^N) = 47.6 ms) × 1024 (*t*_2,max_ (^1^H) = 60.8 ms) complex points, an interscan delay of 0.8 s, and 12 scans per increment were measured. The data was zero–filled to 2048 points in the direct proton dimension and to 256 points in the ^15^N–dimension. Processing was done with a shifted cosine window function for both dimensions. Compound **13** titration series ranged over concentrations of 0, 80, 100, 200, 300, 600, 1000, and 2000 μM; compound **1** was titrated with concentrations of 0, 80, 100, 200, 300, 600, and 1000 μM; compounds **3**, **10**, and **11** were studied at concentrations of 0, 200, 500, 1000, 2000, and 3000 μM and all of the samples contained 80 μM of PIN1 protein and were measured in PIN1 buffer at 298 K. The *T*_1ρ_ relaxation experiments were measured at 298 K with 32768 (*t*_max_ (^1^H) = 1704 ms) complex points, an interscan delay of 2 s and 512 scans per increment. The spinlock times were set at 10 and 400 ms. The STD experiments were measured at 298 K with 16,384 (*t*_max_ (^1^H) = 974 ms) complex points and 512 scans per increment. The on-resonance saturation pulse was set at 0 ppm, and the off-resonance saturation pulse was set to 40 ppm with a saturation time of 1 s.

Thorlabs L450P1600MM, a diode laser emitting at 450 nm. The laser light was coupled (using appropriate coupling optics) into an optical fiber (Thorlabs, FG950UEC) of length 10 m and a diameter of 0.95 mm. The light power output by the laser diode is 1.6 W, and the light power measured at the optic fiber output is 1.0 W due to loss during laser diode–optic fiber coupling.

The HPLC for the sample transfer was a Hitachi L-2200 with an autosampler L-2130. The flow for the sample transfer was set to 1 mL per minute, and the transfer line was a PEEK tubing with an internal diameter of 0.007 inches. The flow was switched using an E.A. 6 Port Valve 7066 interfaced with the HPLC through a TTL analogue to digital converter SS420x from Scientific Software, Inc., and controlled from the Hitachi software LaChrom Elite. The HPLC was in communication with Topspin through an in-house Python script and an in-house built TTL communication system.

The flow cell was 3D printed, the design was done with the software FreeCAD, and the 3D printer was a Projet MJP 2500. The inner tube was a borosilicate tube open at both ends with outer diameter = 3.6 mm and inner diameter = 2.4 mm, purchased from Hilgenberg; the outer tube was a classical 5 mm NMR tube from Wilmad.

## Supporting Information Available

Table of the known photo-CIDNP active compounds; irradiation time dependence of the photo-CIDNP screening experiment; connector for optic fiber details; examples of non-binders’ photo-CIDNP screening spectra; list of hits; quantification of hits polarization ratio and chemical shift perturbations; mapping of the CSPs on the 3D structure of PIN1; HSQC spectra of PIN1 ± compound 15; *K*_d_ fits of selected hits; relaxation and STD NMR screening experiments; sample dilution in the flow cell (PDF)
