# Supplementary material for: Ultrafast Fragment Screening Using Photo-Hyperpolarized (CIDNP) NMR
Source: J Am Chem Soc. 2023 May 25;145(22):12066–80. doi: 10.1021/jacs.3c01392 (PMC10251509; doi:10.1021/jacs.3c01392)
Supplement: Supplementary file 1 — ja3c01392_si_001.pdf [file ja3c01392_si_001.pdf]

Supplementary information to “Ultrafast fragment screening using photo-hyperpolarized (CIDNP) NMR”

*Felix Torres[1,2], Matthias Bütikofer[1], Gabriela R. Stadler[1], Alois Renn[1], Harindranath Kadavath[1,3], Raitis Bobrovs[4], Kristaps Jaudzems[4], Roland Riek[1]**

[1] ETH, Swiss Federal Institute of Technology, Laboratory of Physical Chemistry, Vladimir-Prelog-Weg 2, CH-8093 Zürich, Switzerland
[2] NexMR GmbH, Wiesenstrasse 10A, 8952 Schlieren, Switzerland
[3] St. Jude Children's Research Hospital, 262 Danny Thomas Place, Memphis, TN, 38105-3678 USA
[4] Latvian Institute for Organic Synthesis, Aizkraukles street 21, LV-1006, Riga, Latvia

*Correspondence: roland.riek@phys.chem.ethz.ch

Table S1: Principal photo-CIDNP active known molecules. *The non-exhaustive list of used photosensitizers is reported as flavin mononucleotide (FMN), bipyridyl (BIPY), fluorescein (FLUO), Atto Thio 12 (AT12), 3,3',4,4'-tetracarboxy-benzophenone (TCBP).*

MOLECULE	DYE
**TRYPTOPHAN**	FMN[a], BIPY, FLUO[f], AT12[g], TCBP[e]
**NAC-TRYPTOPHAN**	FMN[a], TCBP[e]
**1-METHYL-TRYPTOPHAN**	FMN[a]
**INDOLE**	FMN[a]
**NAC-SEROTONIN**	FMN[a]
**METHOXY-TRYPTAMINE**	FMN[a]
**TYROSINE**	FMN[a], BIPY, FLUO[f], AT12[g], TCBP[e]
**3-NO_2-TYROSINE**	FMN[a]
**3-F-TYROSINE**	FMN[a]
**3-AMINO-TYROSINE**	FMN[a]
**NAC-TYROSINE**	FMN[a], TCBP[e]
**HISTIDINE**	FMN[c], TCBP[e]
**NAC-HISTIDINE**	FMN[a], BIPY[d], TCBP[e]
**1-METHYL-HISTIDINE**	FMN[a]
**METHIONINE**	FMN[a]
**ADENINE**	FMN[b,c]
**GUANINE**	FMN[b,c]
**3-METHYL-CYTOSINE**	FMN[b]
**5-METHYL-CYTOSINE**	FMN[b]
**THYMINE**	FMN[b,c]
**PORPHYRIN**	1,4 benzoquinone[c]
**POLYPHENOL**	FMN[c]
**HYDROXYPYRROLOINDOLE**	FLUO, AT12, TCBP[h]
**TYRAMINE**	FLUO, AT12[i]
**TRYPTAMINE**	FLUO, AT12[i]
**2,3-DIHYDRO-TRYPTOPHAN**	FLUO, AT12[i]
**INDOLEACETIC ACID**	FLUO, AT12[i]
**INDOLEPROPANOIC ACID**	FLUO, AT12[i]
**3-(2-(PIPERAZIN)ETHYL)-INDOLE**	FLUO, AT12[i]

a)Stob et al., Photochem. Photobiol., 1989[1]; b) Kaptein et al., Journal of the Chemical Society-Chemical Communications, 1979[2] c) Hore, Prog. Nuc. Mag. Res. Sp., 1993[3] d) Tsentalovich, J. Phys. Chem. A, 2000[4] e) Saprygina, J. Phys. Chem A, 2014[5] f) Okuno & Cavagnero, J. Phys. Chem. B, 2016[6] g) Sobol et al., Chem. Phys. 2019[7] h) Torres et al., Phys. Chem. Chem. Phys., 2021[8] i) Torres et al., Mag Res, 2021[9]

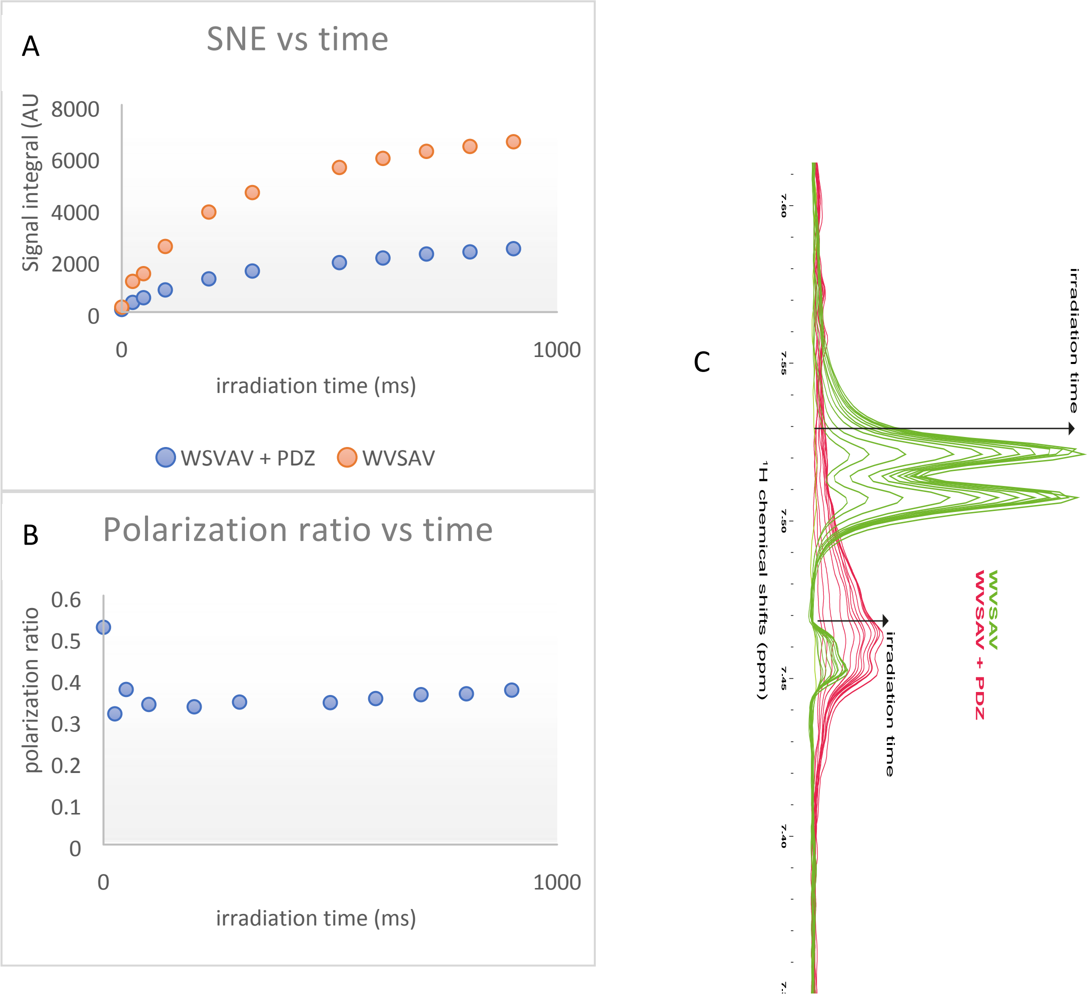

Figure S1: Evolution of the Photo-CIDNP NMR signal with different irradiation time. *A) Signal to noise enhancement (SNE) build-up curve for different irradiation times (ms) at 450 nm and 1W. B) Polarization ratio evolution with irradiation time C) Photo-CIDNP NMR spectra of the WVSAV in green and the WVSAV peptide in the presence of PDZ domain in red. The overlay shows the spectra evolution when the irradiation time prior to radio frequency pulses increases with times 0, 25, 50, 100, 200, 300, 500, 600, 700, 800, 900 ms. The concentration of the WVSAV peptide is 50 µM in both samples (green and red) and the concentration of PDZ2 domain is 50 µM in the sample containing target (red).*

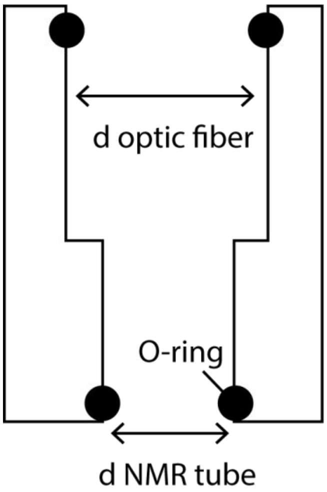

Figure S2: Connector for optic fiber with NMR tube. *In our case the optic fiber diameter (comprising the protective layer) was ca 5 mm and the NMR tubes 3 mm. The protective layer surrounding the optic fiber is then removed at the level of the tube entrance and the optic fiber and is 1 mm.*

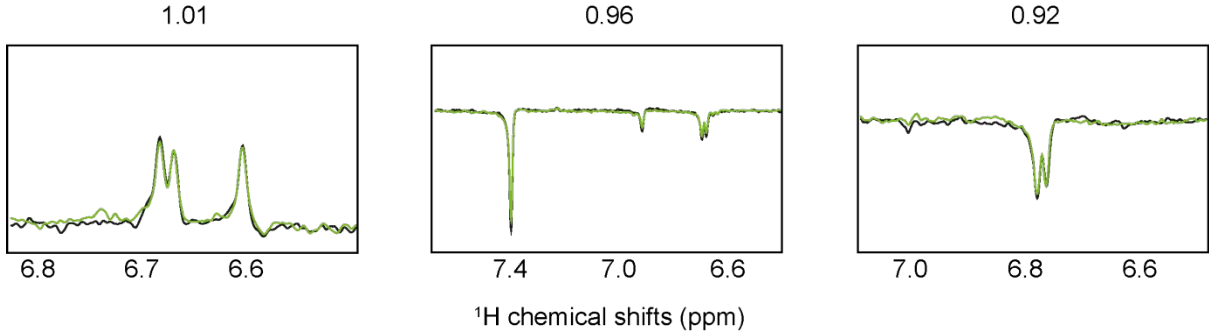

Figure S3: Exemplary non-hits spectra. *The black lines are the photo-CIDNP NMR spectra of small molecule alone (50 µM) and the green lines are the photo-CIDNP NMR spectra of small molecule in the presence of PIN1 protein (50 and 25 µM, respectively). The polarization ratios are provided above each spectrum.*

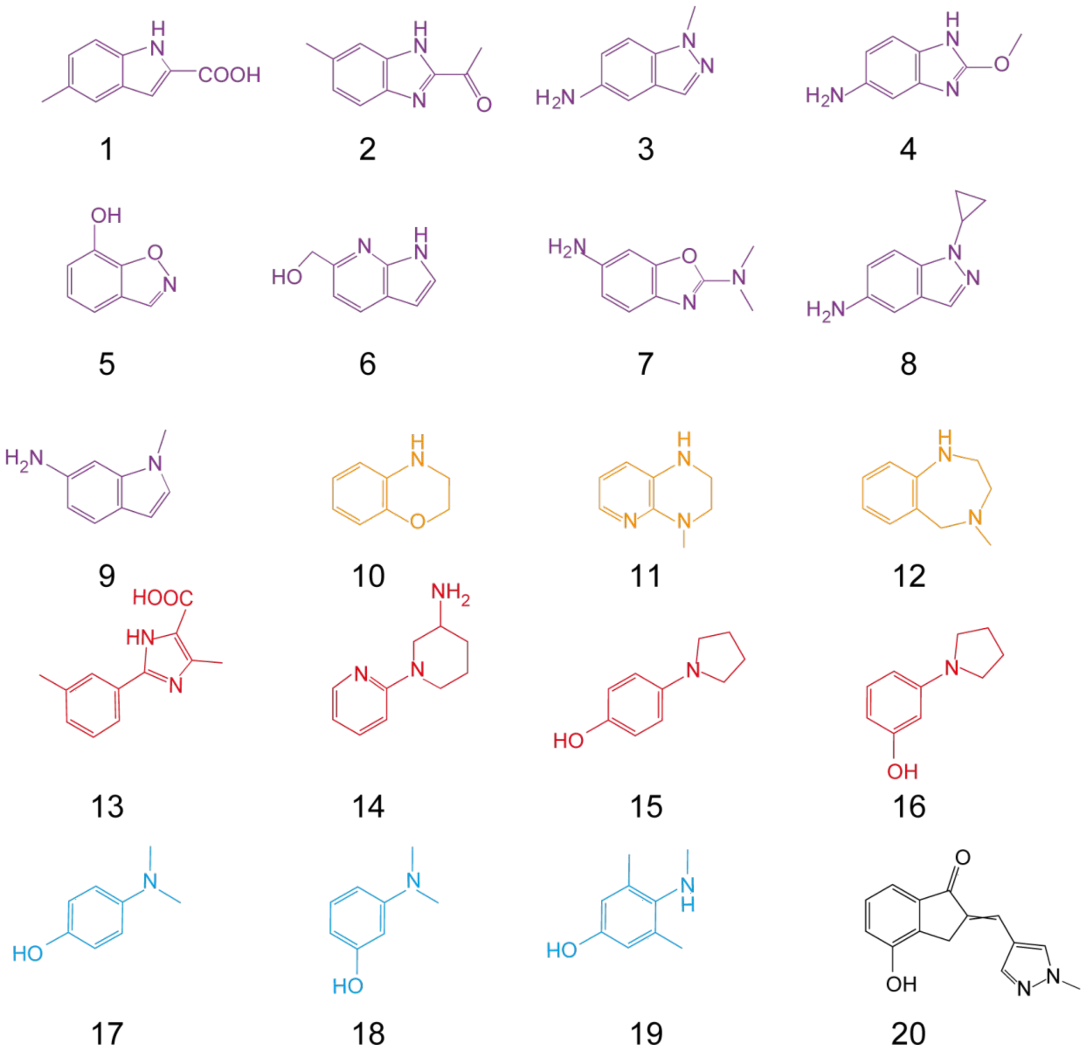

Figure S4: Chemical structures of the fragments showing interaction with PIN1 (hits) through signal reduction of the photo-CIDNP NMR signal during the screening of the NMhare1.0 library against PIN1. *The color code represents the compounds clusters according to structural similarity.*

Table S2: Photo-CIDNP polarization ratios of the different hits (Γ_{PL}/Γ_L). The median chemical shift perturbation observed by [^{15}N-^{1}H]-HSQC is also reported. The median CSP was calculated only on the signals that were showing CSP.

COMPOUND	POLARIZATION RATIO	MEDIAN CSP
**1**	0.42	0.127
**2**	0.69	0.030
**3**	0.75	0.040
**4**	0.22	0.042
**5**	0.28	0.038
**6**	0.64	0.031
**7**	0.79	0.031
**8**	0.34	0.042
**9**	0.42	0.036
**10**	0.38	0.035
**11**	0.53	0.040
**12**	0.45	0.031
**13**	0.38	0.119
**14**	0.63	0.033
**15**	0.23	X
**16**	0.32	0.038
**17**	0.08	X
**18**	0.06	0.035
**19**	0.15	0.032
**20**	0.76	0.031

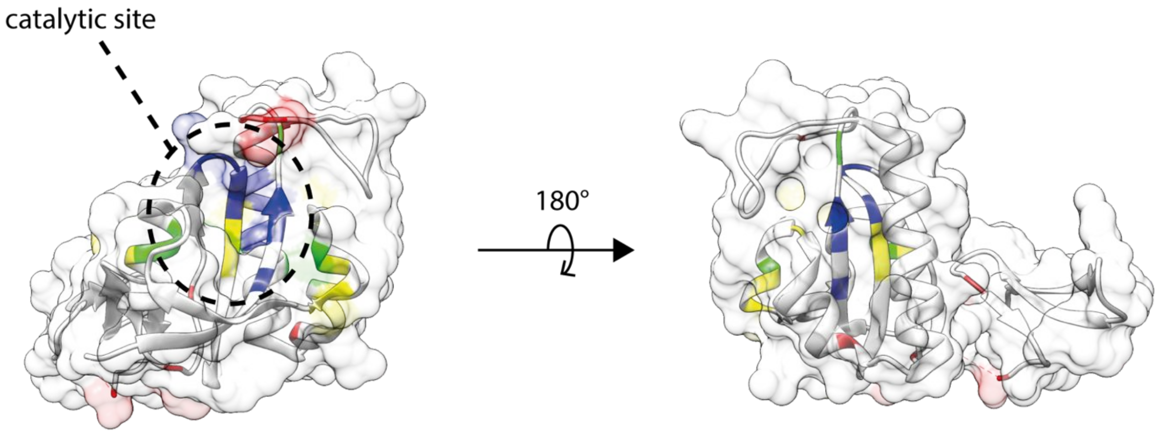

Figure S5: Mapping of the chemical shift perturbations (CSPs) observed for the different hits from the PIN1 screening. *The surface labeled in blue corresponds to the residues for which CSP is observed upon binding of known PIN1-binder, compound 1, and newly discovered hits; The surface labeled in yellow corresponds to the residues for which CSP is observed upon binding of known PIN1-binder, compound 13, and newly discovered hits; The surface labeled in green corresponds to the residues for which CSP is observed for both known PIN1-binders, compounds 1 and 13, and for newly discovered hits; The surface labeled in blue corresponds to the residues for which CSP is observed upon binding of newly discovered hits only.*

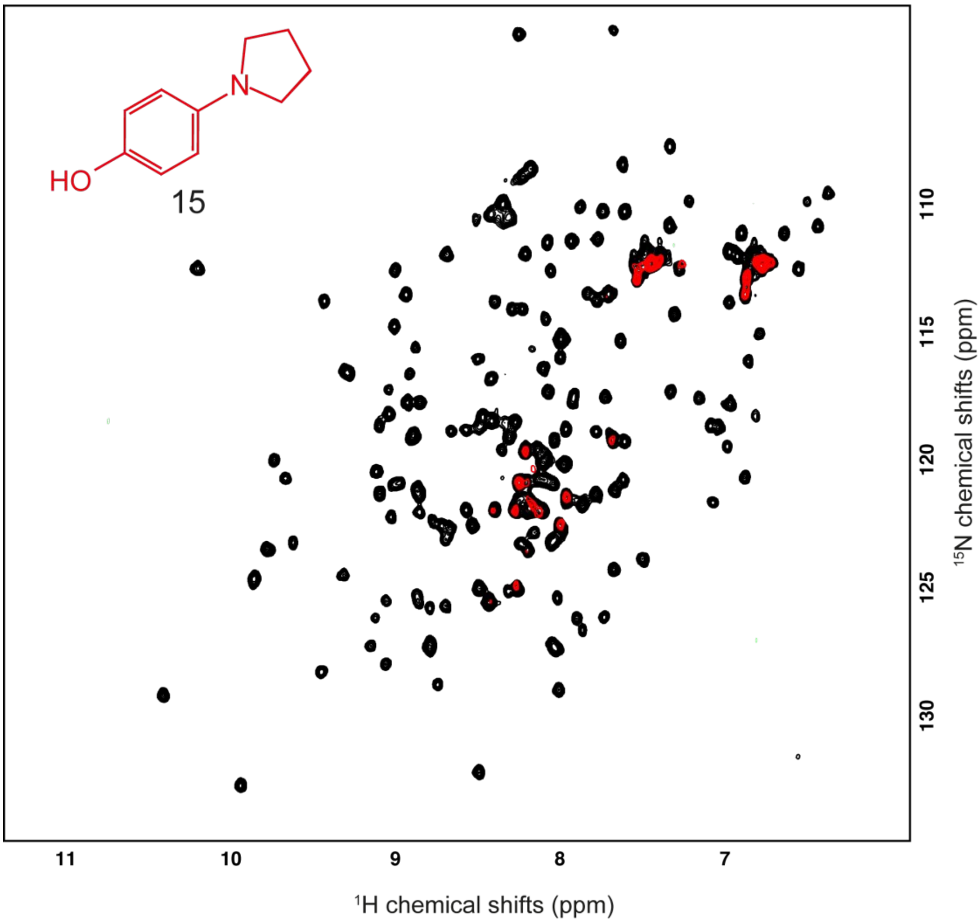

Figure S6: [^{15}N,^{1}H]-HSQC spectra quality deterioration upon addition of compound 15. *Superposition of the reference: [^{15}N,^{1}H]-HSQC spectrum of ^{15}N-labeled PIN1 (black) with the corresponding spectrum in presence of compound 15 (red). Most of the cross peaks got lost indicating the loss of the monomeric well folded state of PIN1. The PIN1 concentration is 50 µM and compound 15 concentration is 200 µM.*

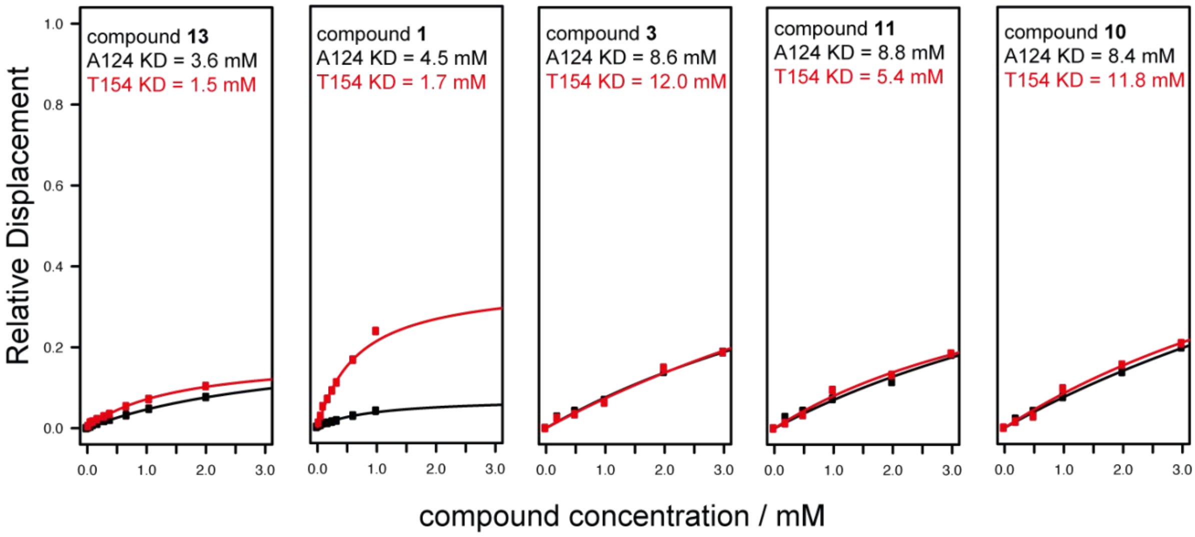

Figure S7: Chemical shift perturbation-based Kd determination of individual compounds binding to PIN1. *The chemical shift displacement of the ${}^{15}N$-${}^{1}H$ moieties of A124 (black) and T154 (red) PIN1 (y-axis) in dependence on the compound concentration (on the x-axis) are indicated for individual compounds 13, 1, 3, 11, and 10. The fits to the titrations are highlighted by a line and the K_d extracted is given in mM. Compound **13** titration series ranges 0, 80, 100, 200, 300, 600, 1000, and 2000 µM concentration; compound **1** was titrated with 0, 80, 100, 200, 300, 600, and 1000 µM compound **1** concentration; compounds **3**, **10**, and **11** range 0, 200, 500, 1000, 2000, 3000 µM concentration and all the samples contained 80 µM of PIN1 protein and were measured in PIN1 buffer at 298K.*

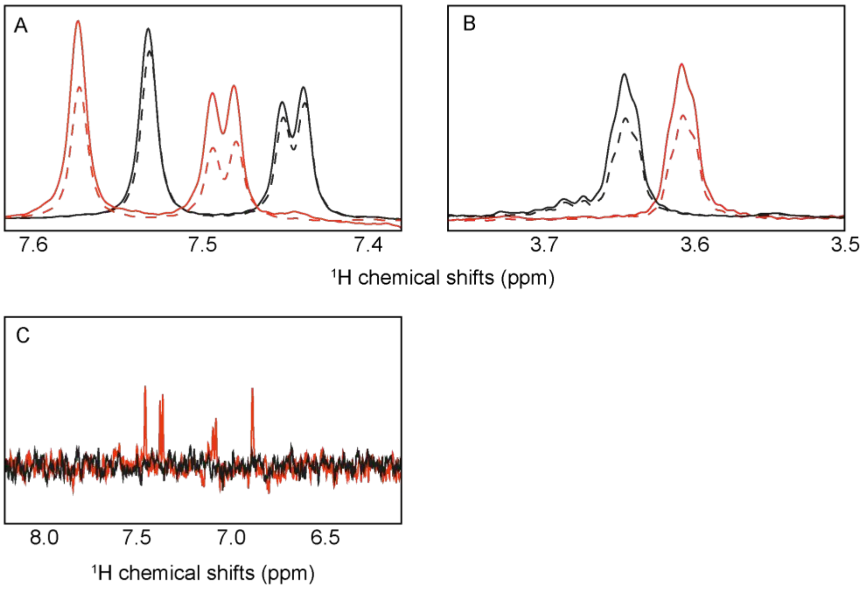

Figure S8: $T_{1\rho}$ and STD NMR screening spectra of compound 1 and 11. *A) $T_{1\rho}$ spectra of compound **1** with sample composition acquired for 200 µM ligand (black lines) and 200 µM ligand and 20 µM PIN1 protein (red lines), the solid line spectra were recorded after 10 ms spinlock and the dashed line spectra were recorded after 400 ms spinlock. B) $T_{1\rho}$ spectra of compound **11** with sample composition acquired for 200 µM ligand (black lines) and 200 µM ligand and 20 µM PIN1 protein (red lines), the solid line spectra were recorded after 10 ms spinlock and the dashed line spectra were recorded after 400 ms spinlock. C) Saturation transfer difference (STD) NMR spectra of compound **1** (200 µM) in the presence (red lines) and absence (black lines) of PIN1 protein (20 µM).*

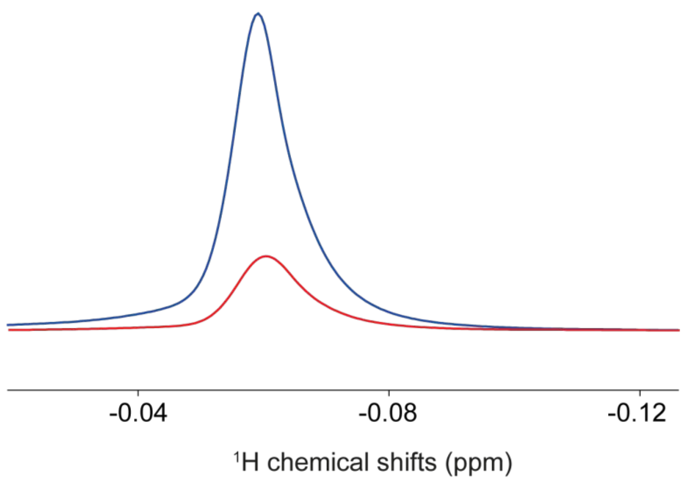

Figure S9: Comparison of the NMR 1H 1D spectra of DSS in a 3 mm tube and in the designed flowcell. *The ratio of the integrals from the signal in 3 mm tube (blue line) and the signal in the design flow cell (red line) gives the value 0.24 (619357/2576446), i.e., a signal-to-noise reduction of ca 4-fold. Each spectrum was measured with 16k points (970 ms) in the direct dimension and a number of scans of 16 with a recycling delay of 3 seconds.*

(1) Stob, S.; Kaptein, R. Photo-Cidnp of the Amino-Acids. *Photochem Photobiol* **1989**, *49* (5), 565-577. DOI: DOI 10.1111/j.1751-1097.1989.tb08425.x.
(2) Kaptein, R.; Nicolay, K.; Dijkstra, K. Photo-Cidnp in Nucleic-Acid Bases and Nucleotides. *Journal of the Chemical Society-Chemical Communications* **1979**, (23), 1092-1094. DOI: DOI 10.1039/c39790001092.
(3) Hore, P. J.; Broadhurst, R. W. Photo-Cidnp of Biopolymers. *Prog Nucl Mag Res Sp* **1993**, *25*, 345-402.
(4) Tsentalovich, Y. P.; Morozova, O. B.; Yurkovskaya, A. V.; Hore, P. J.; Sagdeev, R. Z. Time-resolved CIDNP and laser flash photolysis study of the photoreactions of N-acetyl histidine with 2,2 '-dipyridyl in aqueous solution. *Journal of Physical Chemistry A* **2000**, *104* (30), 6912-6916. DOI: 10.1021/jp000019o.
(5) Saprygina, N. N.; Morozova, O. B.; Grampp, G.; Yurkovskaya, A. V. Effect of amino group charge on the photooxidation kinetics of aromatic amino acids. *J Phys Chem A* **2014**, *118* (2), 339-349. DOI: 10.1021/jp4097919.
(6) Okuno, Y.; Cavagnero, S. Fluorescein: A Photo-CIDNP Sensitizer Enabling Hypersensitive NMR Data Collection in Liquids at Low Micromolar Concentration (vol 120, pg 715, 2016). *J Phys Chem B* **2016**, *120* (48), 12416-12416. DOI: 10.1021/acs.jpcb.6b11197.
(7) Sobol, A.; Torres, F.; Aicher, A.; Renn, A.; Riek, R. Atto Thio 12 as a promising dye for photo-CIDNP. *J Chem Phys* **2019**, *151* (23). DOI: Artn 234201
10.1063/1.5128575.
(8) Torres, F.; Sobol, A.; Greenwald, J.; Renn, A.; Morozova, O.; Yurkovskaya, A.; Riek, R. Molecular features toward high photo-CIDNP hyperpolariztion explored through the oxidocyclization of tryptophan. *Phys Chem Chem Phys* **2021**, *23* (11), 6641-6650. DOI: 10.1039/d0cp06068b.
(9) Torres, F. R., A.; Riek, R. Exploration of the close chemical space of tryptophan and tyrosine reveals importance of hydrophobicity in CW-photo-CIDNP performances. *Magnetic Resonance* **2021**, *2*, 321-329.
